# Aerosol exposure to intermediate size Nipah virus particles induces neurological disease in African green monkeys

**DOI:** 10.1371/journal.pntd.0006978

**Published:** 2018-11-21

**Authors:** Dima A. Hammoud, Margaret R. Lentz, Abigail Lara, Jordan K. Bohannon, Irwin Feuerstein, Louis Huzella, Peter B. Jahrling, Matthew Lackemeyer, Joseph Laux, Oscar Rojas, Philip Sayre, Jeffrey Solomon, Yu Cong, Vincent Munster, Michael R. Holbrook

**Affiliations:** 1 Center for Infectious Disease Imaging, Radiology and Imaging Sciences, Clinical Center, NIH, Bethesda, Maryland, United States of America; 2 NIAID Integrated Research Facility, Ft. Detrick, Frederick, MD, United States of America; 3 Clinical Monitoring Research Program Directorate, Frederick National Laboratory for Cancer Research sponsored by the National Cancer Institute, Ft. Detrick, Frederick, MD, United States of America; 4 Virus Ecology Unit, Laboratory of Virology, Rocky Mountain Laboratories, Hamilton, MT, United States of America; University of Texas Medical Branch, UNITED STATES

## Abstract

Nipah virus (NiV) infection can lead to severe respiratory or neurological disease in humans. Transmission of NiV has been shown to occur through contact with virus contaminated fomites or consumption of contaminated food. Previous results using the African green monkey (AGM) model of NiV infection identified aspects of infection that, while similar to humans, don’t fully recapitulate disease. Previous studies also demonstrate near uniform lethality that is not consistent with human NiV infection. In these studies, aerosol exposure using an intermediate particle size (7μm) was used to mimic potential human exposure by facilitating virus deposition in the upper respiratory tract. Computed tomography evaluation found some animals developed pulmonary parenchymal disease including consolidations, ground-glass opacities, and reactive adenopathy. Despite the lack of neurological signs, magnetic resonance imaging identified distinct brain lesions in three animals, similar to those previously reported in NiV-infected patients. Immunological characterization of tissues collected at necropsy suggested a local pulmonary inflammatory response with increased levels of macrophages in the lung, but a limited neurologic response. These data provide the first clear evidence of neurological involvement in the AGM that recapitulates human disease. With the development of a disease model that is more representative of human disease, these data suggest that NiV infection in the AGM may be appropriate for evaluating therapeutic countermeasures directed at virus-induced neuropathogenesis.

## Author summary

The development of effective therapeutic approaches to the treatment of human diseases requires an understanding of the disease process induced by an infectious agent. Historically the development of medical countermeasures for highly pathogenic viruses required the use of a uniformly lethal animal model. While this approach is useful in some regards, it frequently does not provide a true indication of the disease process. In the work presented here, the approach was to use a virus exposure method that mimicked a potential route of human exposure and used a dose that might be more representative of one a human would receive. Using this method and advanced medical imaging techniques, we were able to demonstrate an extended disease course with mixed respiratory and neurological disease like that seen in humans. This study also found that the response to infection in the lungs was inflammatory and that the disease in the brain was limited despite clear evidence of lesions. These data support the development of animal models that mimic human disease and allow for the identification of potential therapeutic approaches that target the disease process rather than only the virus.

## Introduction

Nipah virus (NiV) is a highly pathogenic paramyxovirus (genus *Henipavirus*) that has been associated with severe respiratory and neurological disease in humans following its emergence in Malaysia in 1998 [1–3]. In humans, NiV infection causes an acute febrile disease with development of atypical pneumonia and respiratory disease that frequently manifests as an Acute Respiratory Distress (ARD)-like disease [4]. Additional signs and symptoms include seizures, areflexia, hypertension and vomiting [5, 6]. Conventional chest X-rays of individuals with respiratory disease identified “ground-glass” opacities [4] suggestive of edema, interstitial thickening or inflammation in the lung parenchyma [7]. Magnetic resonance imaging (MRI) of the brain of individuals with neurological complaints mainly described white matter lesions on T2-weighted and FLAIR images, both subcortical and periventricular in location, with some of the lesions showing restricted diffusion consistent with ischemia [8–11]. Many of those lesions resolved or decreased in size, but with some reports describing eventual appearance of small foci of high signal intensity on T1 with or without decreased signal on susceptibility weighted imaging (SWI) [10, 11], possibly reflecting calcifications developing during convalescence. Long-term neurological complications of the infection, on the other hand, manifest as confluent cortical and subcortical signal abnormalities as well as cortical atrophy [12]. The case fatality rate for NiV infection is around 56% with a higher reported rate in outbreaks in Bangladesh [4, 13].

The reservoir species for NiV are flying fox fruit bats (*Pteropus spp*.) that are common in Malaysia and Bangladesh and through large areas of southern Asia and Australia [14]. During the initial outbreak in Malaysia, pigs were thought to be exposed to NiV through bat excrement or food droppings into pig pens [15]. The pigs became symptomatic with respiratory disease and transmitted the virus to humans likely through droplet or fomite transmission [16]. In Bangladesh, transmission of NiV to humans has been found to occur through consumption of date palm sap that is contaminated by excrement from infected bats [17, 18]. Human-to-human and corpse-to-human transmission has also been documented in Bangladesh and India [19, 20].

Previous studies of NiV infection in the African green (grivet) monkey (AGM) have described evidence of neuropathology [21] that was not apparent in previous work by our group following infection by small-particle aerosol or intratracheal (IT) inoculation [22]. In the case of small particle aerosol and IT inoculation, it is likely that the rapidly progressing, and lethal, respiratory disease prevented development of neurological signs or virus induced neuropathology.

In the work reported here, the objective was to more closely mimic a potential exposure route for humans. We evaluated the pathological response in animals subjected to aerosol exposure with intermediate size (~7 μm) particles with the intent of determining if the location of virus deposition within the respiratory tract impacted disease progression or resolution. Using medical imaging of the lungs and brain, we wanted to assess the AGM for similarity between this model and reported imaging findings in patients with NiV infection along with identification of overt neurological signs of disease based on cage side observations. Additional analyses examined immunological properties in lung and brain tissue, including cytokine levels and infiltrating immune cell populations, that may have been causal in the development of disease. These studies are the first to directly identify neurological abnormalities in the AGM model that are consistent with what has been seen in humans. Furthermore, these studies are the first to report specific immunological changes within the brain and lungs following NiV infection that may be indicative of mechanisms of virus-induced pathogenesis.

## Methods

### Animals

Wild-caught Caribbean origin African green (grivet) monkeys (*Cercopithecus aethiops*) were purchased from PrimGen (Hines, IL). One male and two females were included in each study group. Group sizes were limited due to the imaging requirements in this study. Animals were identified for inclusion based on similarity of size. Animals were group housed prior to being assigned to study and singly housed during the study. At all times animals were provided with appropriate enrichment including, but not limited to, polished steel mirrors and durable toys. Animals were anesthetized in accordance with BSL-4 standard protocols prior to all procedures including inoculation, imaging and collection of blood to minimize stress to the animals. Animals were observed following anesthesia to ensure complete recovery.

Euthanasia criteria were based on a 10-point scoring system that accounted for general signs of disease (e.g. lethargy, not eating, weight loss) and neurological signs (e.g. tremors, paralysis). Secondary criteria such as platelet count (<100,000/dL), coughing and CT examination were also evaluated to help drive decisions for euthanasia based on veterinary discretion, if required.

### Ethics statement

Work with non-human primates was conducted in accordance with an Animal Study Protocol approved by the NIAID Division of Clinical Research Animal Care and Use Committee following recommendations in the Guide for the Care and Use of Laboratory Animals. This institution also accepts as mandatory the Public Health Service policy on Humane Care and Use of Laboratory Animals. All animal work at NIAID was performed in a facility accredited by the Association for the Assessment and Accreditation of Laboratory Animal Care International (AAALACI). All work with non-human primates was done in accordance with the recommendations of the Weatherall Report.

### Virus and cells

The Malaysian strain of NiV that was used in this study was isolated from a fatal human case in 1998 [2]. The documented passage history includes three passages in Vero E6 cells, one passage in Vero cells and two additional passages in Vero E6 cells. The stock virus sequence information has been submitted to GenBank (Accession #KY425646.1) and is consistent with the previously published sequence for this virus (#AF212302).

VeroE6 cells (BEI #NR596) were maintained at 37°C/5% CO_2_ in α-MEM w/GlutaMAX (Gibco) and containing 10% fetal bovine serum (FBS). All work with viable NiV was performed in the BSL-4 facility at the NIAID Integrated Research Facility in Frederick, MD.

### Aerosol exposure

Two groups of three AGMs were exposed to either a low dose (group 1, 100 plaque-forming unit (pfu) target dose (low)) or high dose (group 2, 1000 pfu target dose (high)) dose in a medium-large particle (target 7 μm) aerosol challenge of NiV-Malaysia using a 16 Liter, head-only aerosol exposure chamber. The two cohorts were exposed on consecutive days to accommodate imaging procedures associated with this study. An aerosol management platform (AeroMP, Biaera Technologies, USA) within a Class III biosafety cabinet (Germfree, FL, USA) was used to conduct the aerosol challenge. The aerosol exposure chamber (-0.1” WC) and Class III BSC (-1.0” WC) both maintained negative pressure throughout the entire experiment. Challenge material was thawed, pooled, diluted, and divided into (3) 10 mL aliquots prior to challenge and kept on wet ice. The animals were anesthetized with ketamine (10 mg/kg, IM) and Telzaol (4–6 mg/kg, IM) and received a single, time calculated aerosol challenge dose. Respiration values and breathing frequencies for the AGMs were obtained using a calibrated pneumotachograph mask just prior to the aerosol exposure. Aerosol particles were generated by centered flow tangential aerosol generator (CenTAG, CH Technologies, NJ, USA) operating at 12.0 liter/min (LPM) generator air and 12 LPM dilution air, which produced medium-large particles ranging from 6.5–8.0 μm in size targeting the tracheobronchial and nasopharyngeal regions of the respiratory tract. A 6 LPM siphon vacuum was also used during CENTAG operation to siphon out smaller aerosol particles. An aerodynamic particle-sizing device (Aerodynamic Particle Sizer, TSI, MN, USA) verified particle size with real-time measurements (6.76 μm Mass Median Aerodynamic Diameter; 1.43 Geometric Standard Deviation). Gelatin filter biosamplers (Sartorius Stedim Biotech, Germany) operated at a continuous flow rate of 2.0 LPM and collected aerosol challenge material to determine the aerosol concentration within the exposure chamber. The gelatin filters were diluted in Dulbecco’s Modified Eagle Medium (Lonza, MD, USA) and bovine serum albumin (Sigma-Aldrich, MO, USA). An air wash period of 5 minutes between each aerosol challenge allowed the previously generated particles within the exposure chamber to decay. A presented dose was calculated using the simplified formula D = R x C_exp_ x T_exp_, where D is the presented or estimated inhaled dose (PFU), R is the respiratory minute volume (L/min), C_exp_ is the aerosol concentration (PFU/L), and T_exp_ is the duration of the exposure (min). These formulas have been outlined previously [23]. The low dose group received an average presented dose of 63.68 pfu and the high dose group received an average presented dose of 701.50 pfu (S1 Table).

### Virus titration

Virus stocks and experimental samples were titrated on Vero E6 cells using a plaque assay as previously described [22]. Briefly, 10-fold serial dilutions of test sample were inoculated onto Vero E6 cells in 6-well plates. The cells were overlain with semi-solid 1.25% Avicel (f/c) (FMC Biopolymer) diluted in EMEM (Gibco) with 2% FBS (f/c). The cells were incubated for 3–4 days at 37°C/5% CO_2_ before being fixed and stained with 0.2% crystal violet in neutral buffered formalin (NBF) and plaques enumerated.

### Clinical analyses

Serum chemistries were completed using a General Chemistry 13 standardized analysis panel (Abaxis) and run on an Abaxis Piccolo. Complete blood counts (CBC) from whole blood were obtained using a five-part differential with reticulocytes on a Sysmex XT2000*i*V. Data were analyzed and plotted using Prism (GraphPad Software, LaJolla, CA).

### Imaging

Animals were imaged prior to study start to establish baseline images and then on alternate days from day 2–16 post-exposure and weekly thereafter. Animal subjects underwent imaging on Philips Precedence SPECT/CT and Achieva 3 Tesla magnetic resonance clinical scanners (Philips Healthcare, Cleveland, OH). For Computed Tomography (CT), the subjects were imaged for an average scan acquisition length was 42 seconds. The CT unit is a 16 slice, multi-dimensional clinical scanner (Philips Healthcare, Cleveland, OH, USA). Subjects were immobilized using isoflurane, and positioned on the imaging table in a supine, head out, feet in, and arms up fashion. Two scout images were performed first, followed by a high resolution, breath hold CT scan. CT parameters used for the acquisition included 140 kVp (kiloVolts peak), 300 mAs (milliAmperes * second), a slice thickness of 0.8 mm with 0.40 mm increment, and a matrix size of 512 x 512. Images were reconstructed to a 160 mm field of view resulting in a pixel size of 0.3 mm x 0.3 mm. Quantification of changes in lung volume were performed as previously described [22, 24].

For brain magnetic resonance imaging (MRI) studies, all animals underwent a 65-minute long series of scans using a Pediatric Head/Neck coil. Subjects were intubated, immobilized using isoflurane, and positioned on the scanner bed in a supine fashion. Animals were prepped with an IV catheter in an arm vein before entering the imaging suite and a line pre-charged with Magnevist was used for manual injection followed by a saline bolus to flush the system. Structural, quantitative and functional measures were obtained, and all slices were acquired in the axial plane. Imaging sequences obtained included T_1_- and T_2_-weighted fast field echo (FFE) sequences, magnetization-prepared rapid gradient echo (MPRAGE) and principle of echo shifting (PRES). The FLAIR and T_1_-weighted FFE sequences were performed both pre- and post-contrast.

### Necropsy and histopathology

Necropsies were performed on each subject with tissues collected from the same region in each individual animal. Tissues for histopathology were fixed in 10% NBF for at least 72 hours prior to removal from biocontainment. The tissues were then embedded in paraffin, sectioned, mounted on glass slides and stained for hematoxylin and eosin (H&E). Slides stained with H&E for morphologic assessment were then read by a veterinary pathologist. Slides prepared for immunohistochemistry were treated with xylene to remove paraffin and rehydrated through a graded series of alcohols. The primary antiserum used for staining was a NiV-GP specific antiserum generated in rabbits [25].

### PCR

Evaluation for the presence of viral RNA was performed using a quantitative Real-Time PCR assay (qRT-PCR) that is specific for viral genome, as previously described [26], and identifies sites of viral replication or genomic RNA isolated from virions.

### Cytokine response

The quantification of cytokines in clarified tissue homogenates was performed in duplicate using a 23-plex NHP cytokine assay following the manufacturer’s instructions (Millipore, Burlington, MA) on a FlexMap analysis system (Luminex, Austin, TX). Data were analyzed using Microsoft Excel and Prism 7.

### Immunology

Immune cell populations in the lung and brain were isolated by collagenase digestion as previously described [22]. Briefly, digested tissues were strained through a 100μm strainer and collected by pelleting. Lung cells were resuspended in ACK Lyse (Quality Biologics) to lyse red blood cells and the residual cells pelleted, resuspended in PBS and counted. Brain cell pellets were re-suspended in Percoll stock solution and purified against a Percoll working stock solution. The interface was collected, and the cells washed, resuspended in PBS and counted. The cells were then stained as previously described [22] using a 17-color panel. Data was collected on an LSR Fortessa flow cytometer (BD Biosciences) and analyzed with FlowJo and GraphPad analysis software.

### Statistical analysis

Given the limited sample size (n = 3) of the two groups used in this study and the use of out-bred animals, statistical analysis was not performed. Rather, data are presented for individual animals and compared relative to baseline data where appropriate.

## Results

### Clinical

**Fig 1:**
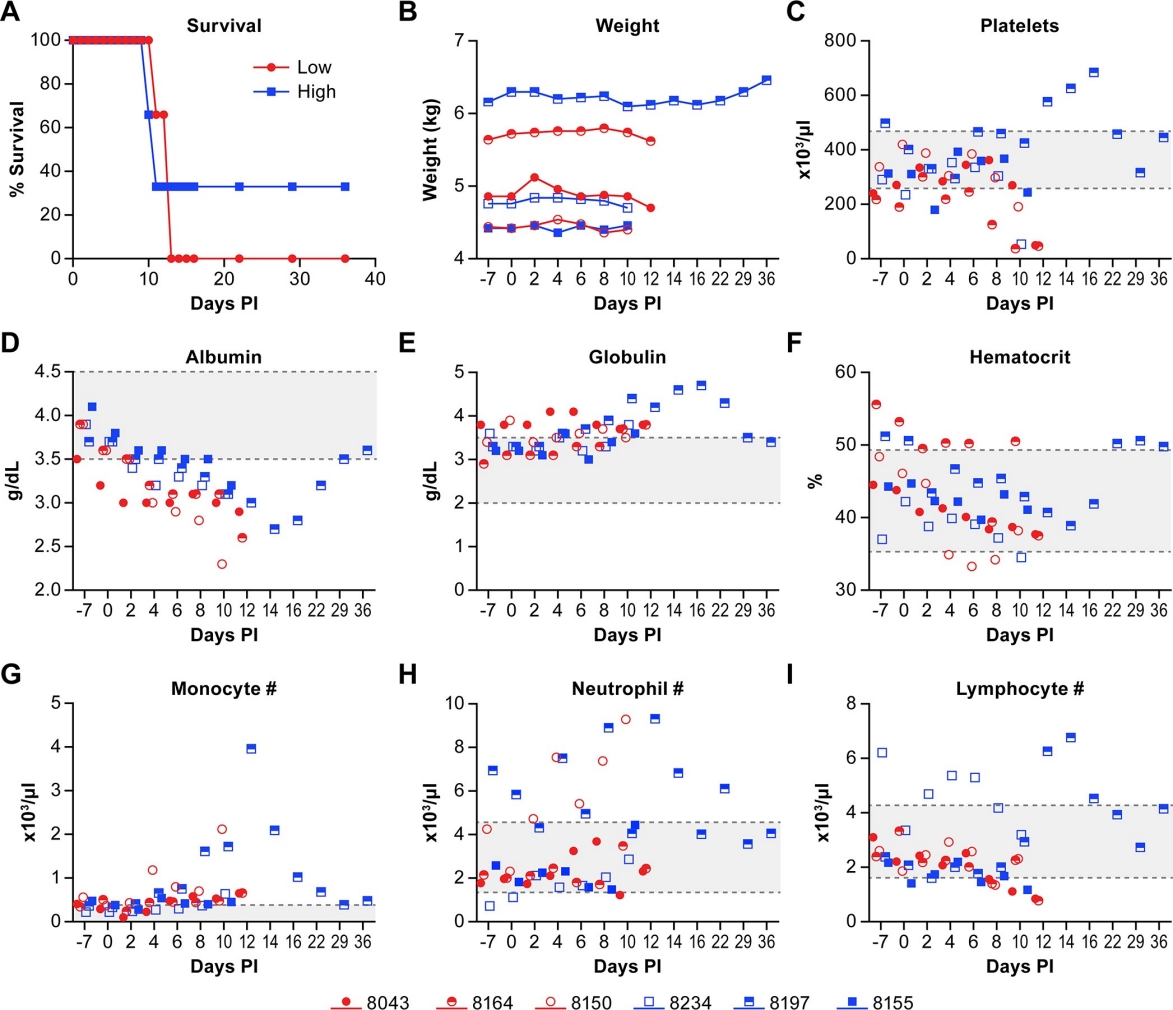
Clinical parameters of NiV infection. Clinical and hematological comparisons between the low (red circles) and high (blue squares) dose groups and individual animals. The color scheme and symbols used in this figure are consistent throughout the study for each individual animal. Animals were evaluated daily for clinical changes in well-being and bleed on alternate days through day 16 post-exposure for weight, chem-13 and CBC analyses. Data are presented here for (A) Survival; (B) Weight; (C) Platelets; (D) Albumin; (E) Globulin; (F) Hematocrit; (G) Number of monocytes per μl; (H) Number of neutrophils per μl; (I) Number of lymphocytes per μl. The gray area between the dashed lines in panels C-I represent the range of average values for Caribbean origin AGMs [40].

An objective of these studies was to determine if the disease process in AGMs following exposure to a virus aerosol that achieved deposition in the mid- to upper respiratory tract [27] was markedly different from IT or small-particle aerosol exposure. Two different dose groups (low-approximately100 pfu target dose; high-approximately 1000 pfu target dose) were used to determine if the amount of virus delivered had an impact on disease progression. The measured particle size for these exposures ranged from 6.10–7.19μm. The calculated exposure dose based on back-titration was 22–1197 pfu (S1 Table). The average survival time (AST) was approximately 12.5 days for the five animals that succumbed to the infection, with one animal surviving (Fig 1A), and there was no clear relationship between the dose received and time of survival. However, the AST in this study was longer than previous reports using IT or small-particle aerosol exposure [21, 22, 28, 29].

Marked weight loss was not noted (Fig 1B) despite animals having a reduced appetite, although there was a slight decrease in some animals late in the disease course. Similar to previously published work, thrombocytopenia was evident in most animals late in the disease stage (Fig 1C). All animals had evidence of decreased albumin and increased globulin levels that returned to normal in the animal that survived (Fig 1D and 1E). Some animals also had anemia (8150, 8234), monocytosis (8150, 8197), neutrophilia (8197, 8150) and/or lymphopenia (8164, 8043, 8155) (Fig 1F–1I), which are consistent with previous reports of NiV infection in this model. There were no clear and consistent differences between the two virus dose groups.

Cage side observations identified lethargy, reduced ration consumption and reduced fecal output as common signs of disease. Some animals developed a cough, an indicator of respiratory disease, but there was no increase in the respiratory rate until the terminal day of disease. None of the animals had overt signs of focal neurological disease.

### Imaging

**Fig 2:**
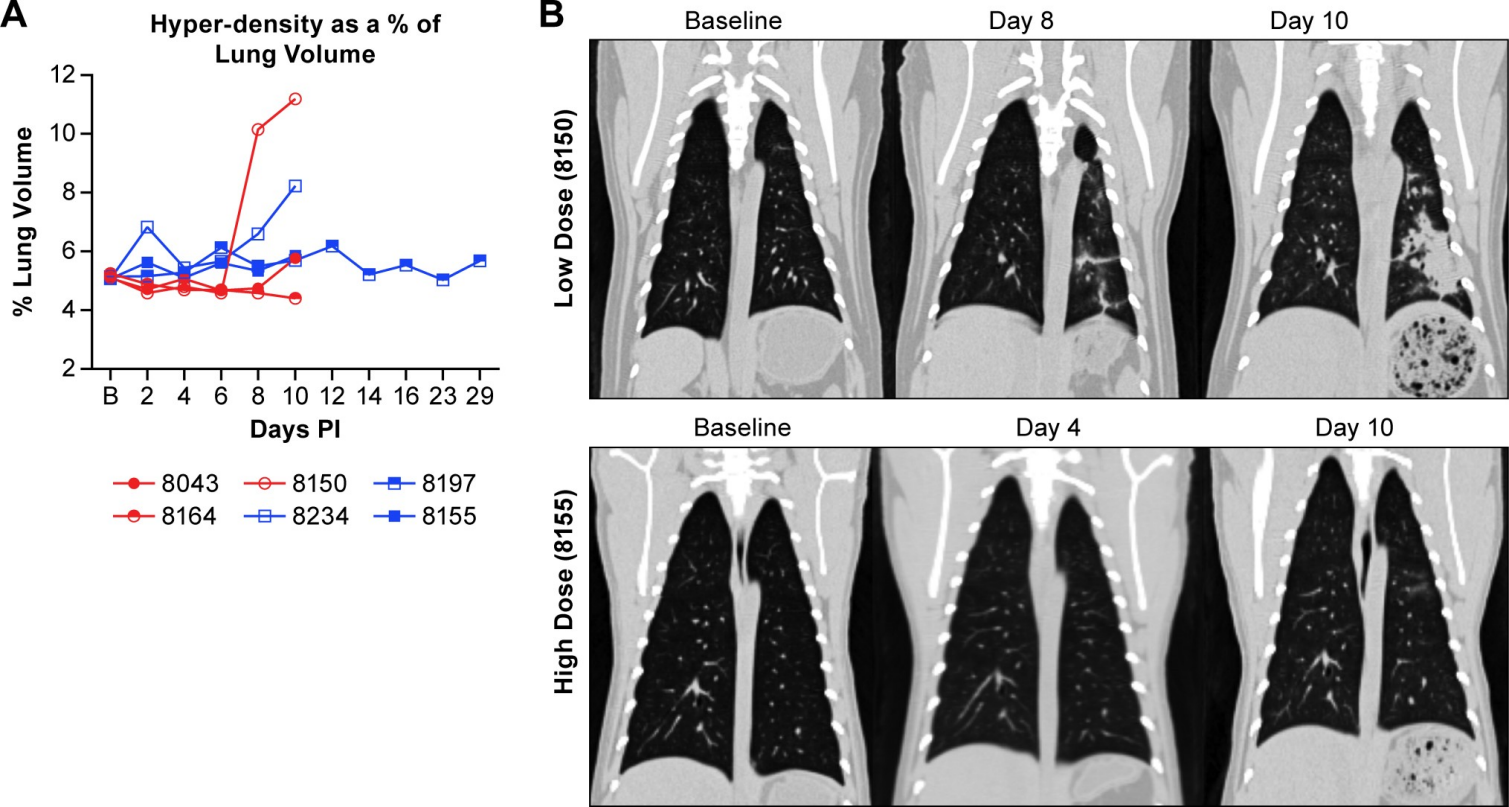
Evaluation of lung volume in NiV infected AGMs. Computed tomography (CT) scans were performed prior to NiV aerosol exposure (Baseline-B) and every second day following. Changes in lung volume (A) were quantified based on mapping of post-exposure images to pre-exposure baseline scans as previously described [22]. Volumetric quantification was calculated based on Hounsfield unit thresholds using histograms to determine threshold values. (B) Examples of CT images from two animals at the specified days post-virus exposure. The top panels demonstrate increased hyperdensity and a decrease in uninvolved lung volume while the lower panels show an animal that had minimal pulmonary changes based on CT.

The use of CT to determine lung pathology and changes of respiratory volume is a critical component of monitoring real-time disease progression in this animal model system. Our CT assessments found that there was no appreciable difference in lung volume in animals infected with the high or low dose of NiV. One animal in the low dose group (8150) developed lung consolidation in the left caudal lobe with patchy infiltrates also noted in the right and left cranial lobes (S2A Table). Areas of ground-glass appearance were seen, as well as air bronchograms within the consolidated lung that resulted in an approximately 11% loss in uninvolved lung volume (Fig 2A). An animal in the high dose group (8234) had approximately 9% loss in lung volume resulting from focal consolidation and ground-glass opacities. In other animals there were also patchy foci of ground-glass opacifications in different locations, some of which resolved or decreased on the last imaging day. Interestingly, animals often had evidence of abnormal lung opacities on days 2 and 4 that resolved before more distinct lesions appeared on days 8 and 10. This is possibly due to an inflammatory response to the initial aerosol insult followed by NiV induced changes later in the disease process. Fig 2B provides examples of the pathology seen in the animal with the most appreciable lung volume loss and one of the animals with minimal pathology.

**Fig 3:**
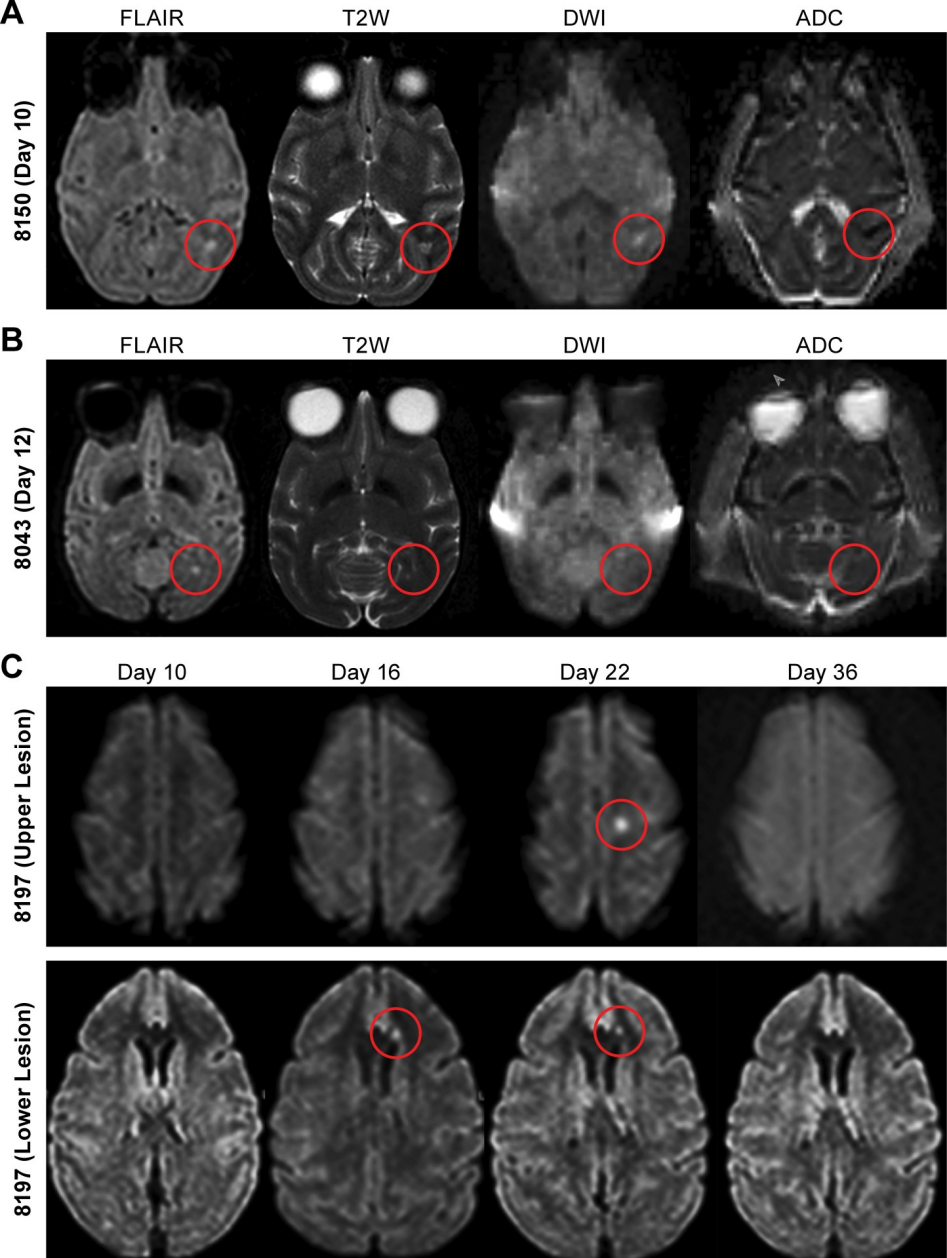
Demonstration of NiV-induced lesion in the brain of AGM. Magnetic resonance imaging (MRI) was performed prior to and at multiple time points following NiV-exposure. (A) MRI scans from animal #8150 obtained at day 10 PI. A small focal area of increased signal intensity is seen in the left temporal subcortical white matter on FLAIR and T2 weighted (T2W) images. This corresponded to increased signal on DWI and decreased signal on ADC (restricted diffusion) suggestive of acute/subacute ischemic focus. (B) similar sequences obtained in animal 8043 at day 12 PI show a small hyperintense focus in the left occipital subcortical white matter on T2 and FLAIR images, however with no evidence of restricted diffusion. (C) FLAIR images obtained on days 10, 16, 22 and 36 PI in animal 8197 show focal hyperintensity in the left centrum semiovale, appearing on Day 22 (upper panel) and another focal hyperintensity in the left frontal periventricular white matter, appearing on day 16 (lower panel). Along with a third similar lesion in the left occipital region that is not visible in these slices, none of the lesions was associated with restricted diffusion or enhancement and all three lesions resolved by day 36 PI.

Magnetic resonance imaging of animals included in this study showed abnormalities reminiscent of human pathology in three out of six animals (50%), including in one animal which survived the infection. In one animal (#8150), two lesions were seen, one in the left occipital and one in the right temporal subcortical white matter on day 10 (terminal day) (Fig 3A showing the left occipital lesion). Both lesions showed associated increased signal on diffusion-weighted imaging (DWI) and decreased signal on apparent diffusion coefficient (ADC) maps (restricted diffusion). A second animal (#8043) showed two small hyperintense lesions with no restricted diffusion, one in the right external capsule and one in the left occipital white matter on day 12 (terminal day) (Fig 3B showing the left occipital lesion). In the survivor (#8197), three small focal high signal intensity lesions were seen on FLAIR and T2-weighted images: in the left centrum semiovale on day 22 (Fig 3C-upper panel), in the left frontal subcortical white matter on day 16 (Fig 3C-lower panel) and in the left occipital region on day 16 post-infection. None of those lesions showed restricted diffusion and all resolved by day 36 (Fig 3C, top and bottom panels). None of the lesions appeared to enhance after contrast administration. Unlike in patients, none of the lesions eventually showed increased signal on T1, probably due to lack of extended follow up in the infected animals and the fact that only one animal survived disease.

### Gross pathology

Pathological evaluations of animals at necropsy were generally consistent with previous findings [21, 22, 29]. None of the animals had obvious brain disease manifestations on gross pathology while some animals had congestion and hemorrhage in the lungs with two animals having developed interstitial pneumonia (S2B Table). Some animals had evidence of petechial hemorrhage on their skin (8234, 8197, 8155) and on the mucosa of the urinary bladder (8164, 8150, 8234) and two had evidence of hemorrhage and/or edema in the mediastinum (8043, 8155), but no gross damage to the myocardium. Examined lymph nodes were frequently edematous and congested and the spleen was enlarged or turgid in three (8164, 8043, 8234) of the six animals.

### Histology

**Fig 4:**
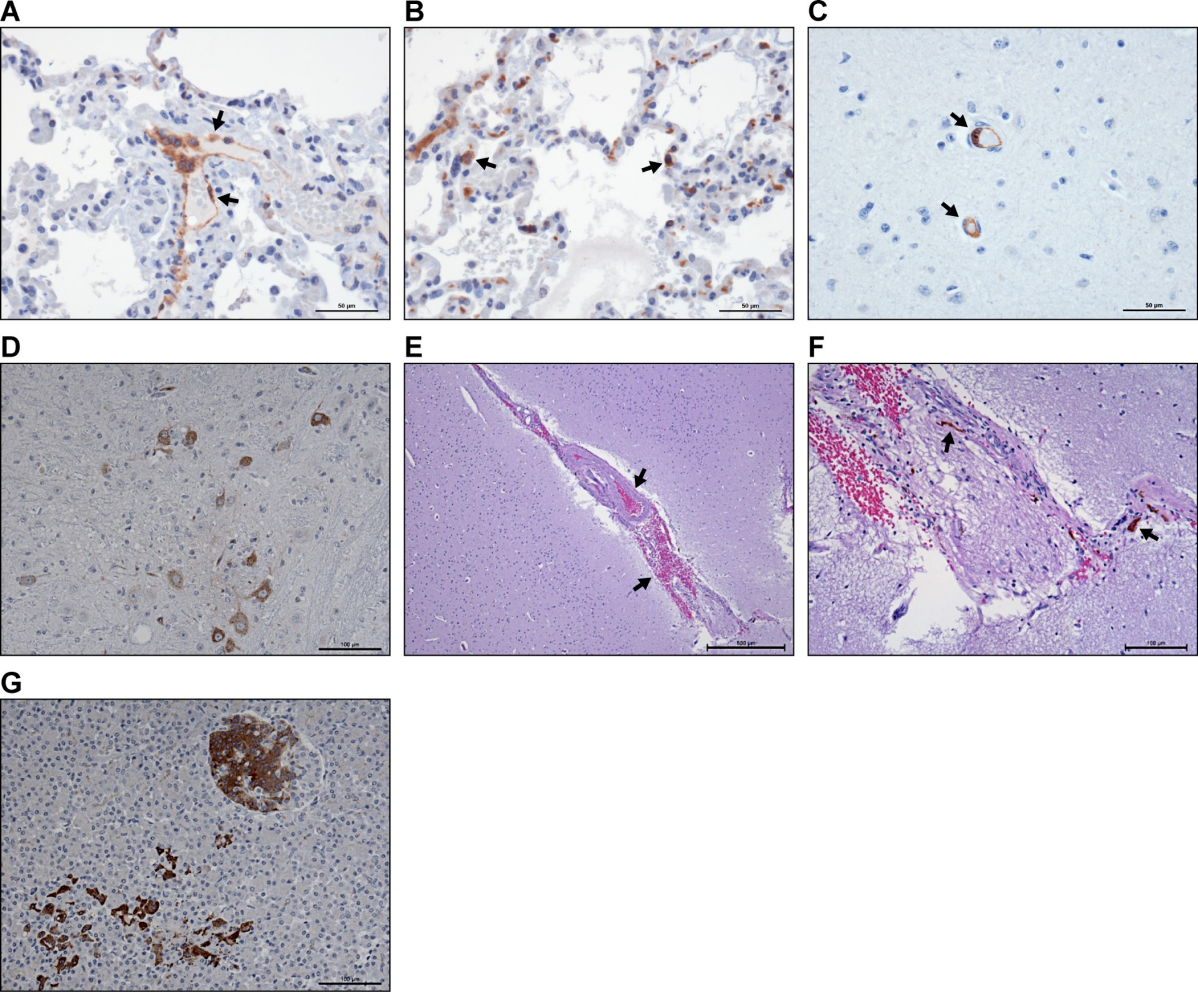
Histopathological assessment of select tissues from AGM following NiV exposure. Immunohistochemical analysis of tissues from NiV infected animals identified viral antigen in several different cell types, but was more prominent in (A) endothelial cells and histiocytes. In the lung, viral antigen was also found in (B) pneumocytes. In the brain, (C) virus was generally found in endothelial cells (C), with antigen found occasionally in neurons (D), but only in foci rather than broadly distributed. Following identification of a lesion in the brain by MRI (see Fig 3C-lower panel), histological assessment of the hyperintense lesion (E and F) identified a microhemorrhage with hemosiderin laden macrophages apparent, but no evidence of viral antigen. Viral antigen was also identified in the pancreas where degeneration of the islets was evident (G). Arrows in (A and (C) indicate viral antigen in endothelial cells. Arrows in B indicate viral antigen in pneumocytes. Arrows in (E) indicate areas of hemorrhage. Arrows in (F) indicate hemosiderin laden macrophages. Scale bars for panels (A-C) are 50 μm, for panels (D) (F) and (G) are 100 μm; for panel (D) is; and for panel (E) is 500 μm.

Histological evaluations of tissues collected at necropsy were consistent with previously described findings. In brief, most animals had varying degrees of vasculitis in examined tissues with evidence of multinucleated cells and viral antigen found in epithelial cells, endothelial cells and histiocytes (Fig 4A and S2C Table). In the lungs, viral antigen was also found in pneumocytes (Fig 4B). Unlike our previous work that used IT or small particle aerosol infection [22], congestion in the lung in this study was diffuse or mild, with hemorrhage or edema less commonly seen. There were no consistent gross abnormalities seen in the brain with only one animal (#8164) displaying clear evidence of acute disease where meningitis with hemorrhage was identified in the brainstem (S2C Table). When viral antigen was noted in the brain, it was typically in the endothelial cells (Fig 4C) with occasional antigen identified in neurons (Fig 4D). The lesion identified by MRI in the surviving animal (Fig 3C) was located and identified as a microhemorrhage with no evidence of viral antigen (Fig 4E and 4F). The lesions identified in the non-surviving animals were too small to be identified histologically.

In this study pancreatitis was identified as a significant pathological feature in four (8043, 8164, 8150, 8155) of the six animals included in the study. All three of the animals in the low dose group had multifocal degeneration of the islets and viral antigen found to varying degrees within this organ (Fig 4G and S2C Table). Glucose levels for these animals were not outside of normal ranges and additional markers of pancreatic function were not evaluated.

### RNA and virus titers

**Fig 5:**
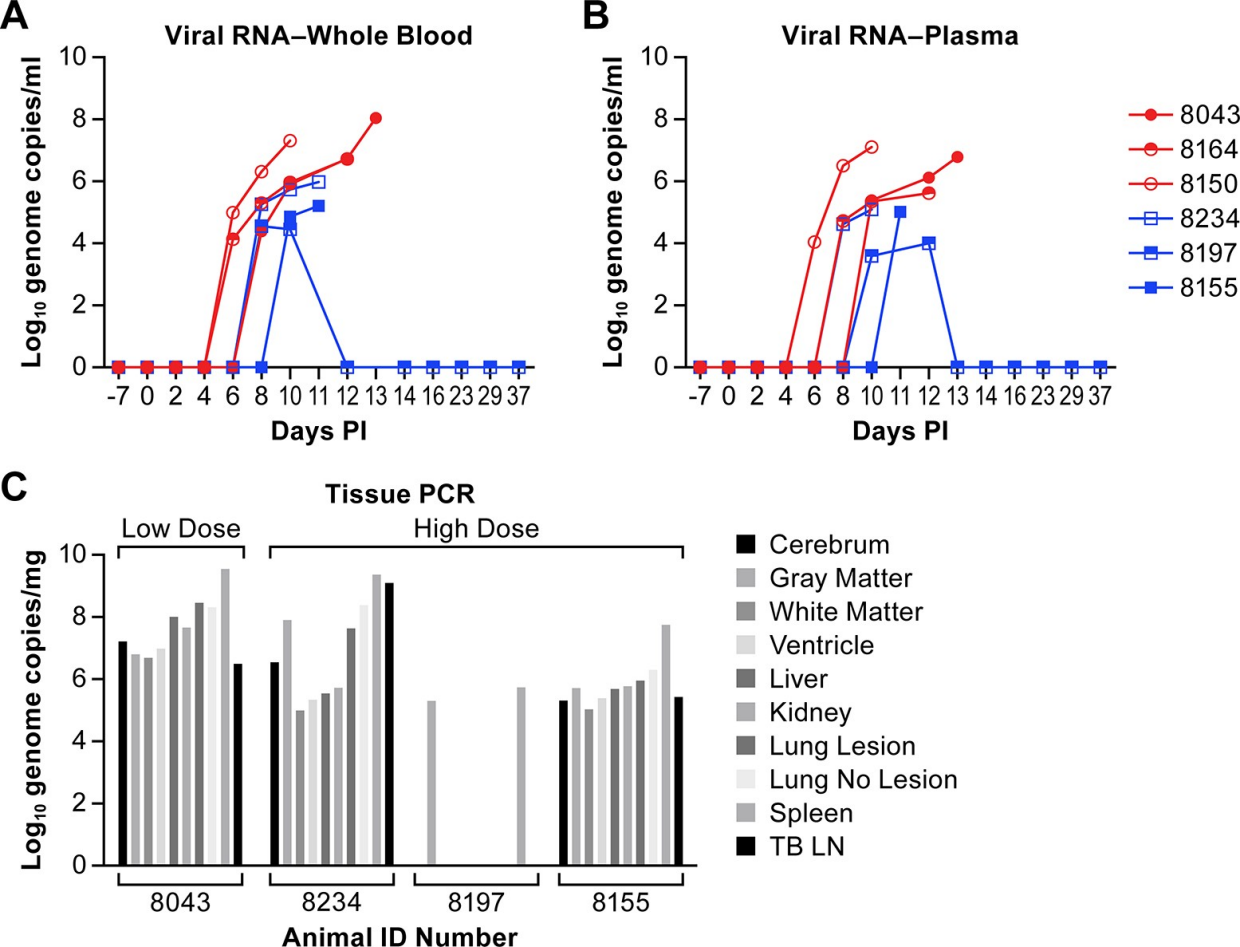
Determination of viremia and viral RNA distribution. Whole blood (A) and plasma (B) were collected and viral RNA levels quantified using a viral genome specific RT-PCR assay that targets a genome intergenic region [26]. Genomic titers for individual animals in the low dose (red circles) and high dose (blue squares). (C) The level of virus RNA was determined in multiple tissues from four of the six animals included in the study. The limit of detection for this PCR assay is approximately 6x10^3^ genomes per mL.

Viral genomic RNA levels were determined in plasma and whole blood over the course of disease and at necropsy. In two animals from the low dose group, viral RNA levels of 4–5 log_10_ genome copies were evident in whole blood at day 6, while in the remaining animals viral RNA was not evident until days 6–8 (Fig 5A). The profile in plasma was similar to whole blood in all animals (Fig 5B). The animal that survived infection had lower peak viral genome levels than the other five animals in both whole blood and plasma at 4.56 log_10_ and 4.0 log_10_, respectively, suggesting a correlate of potential survival.

To demonstrate the presence of NiV RNA in tissues collected at necropsy, viral genomic RNA was quantified. Samples from two animals that succumbed to the infection (8164 and 8150) were not tested due to degradation of the tissues prior to necropsy. As with previous studies, viral RNA was found in all tested tissues collected from animals in the terminal acute phase of disease (Fig 5C). However, these animals were not perfused prior to tissue collection and viral RNA levels in the blood may contribute to the levels of viral RNA identified in the tissues. In the one surviving animal, viral RNA was found only in the gray matter of the brain and in the spleen.

### Tissue cytokine response

To determine the impact of virus infection on tissue immune response, homogenized tissues collected at necropsy were tested for cytokine expression using a multi-plex assay format. This assay included an array of inflammatory and stimulatory cytokines that may be induced during viral infection. Also included were several chemokines associated with migration of both lymphocytes and antigen presenting cells (APCs). In general, there was no evidence of a systemic inflammatory response; rather, there were tissue specific changes that appear to represent a local response to infection. Tissues from two uninfected control animals, one male and one female, were tested independently and the cytokine response data for individual tissues is included in S1 Fig. While the sample size for control animals is small, these data can provide an approximation of “normal” cytokine levels in the collected tissues.

**Fig 6:**
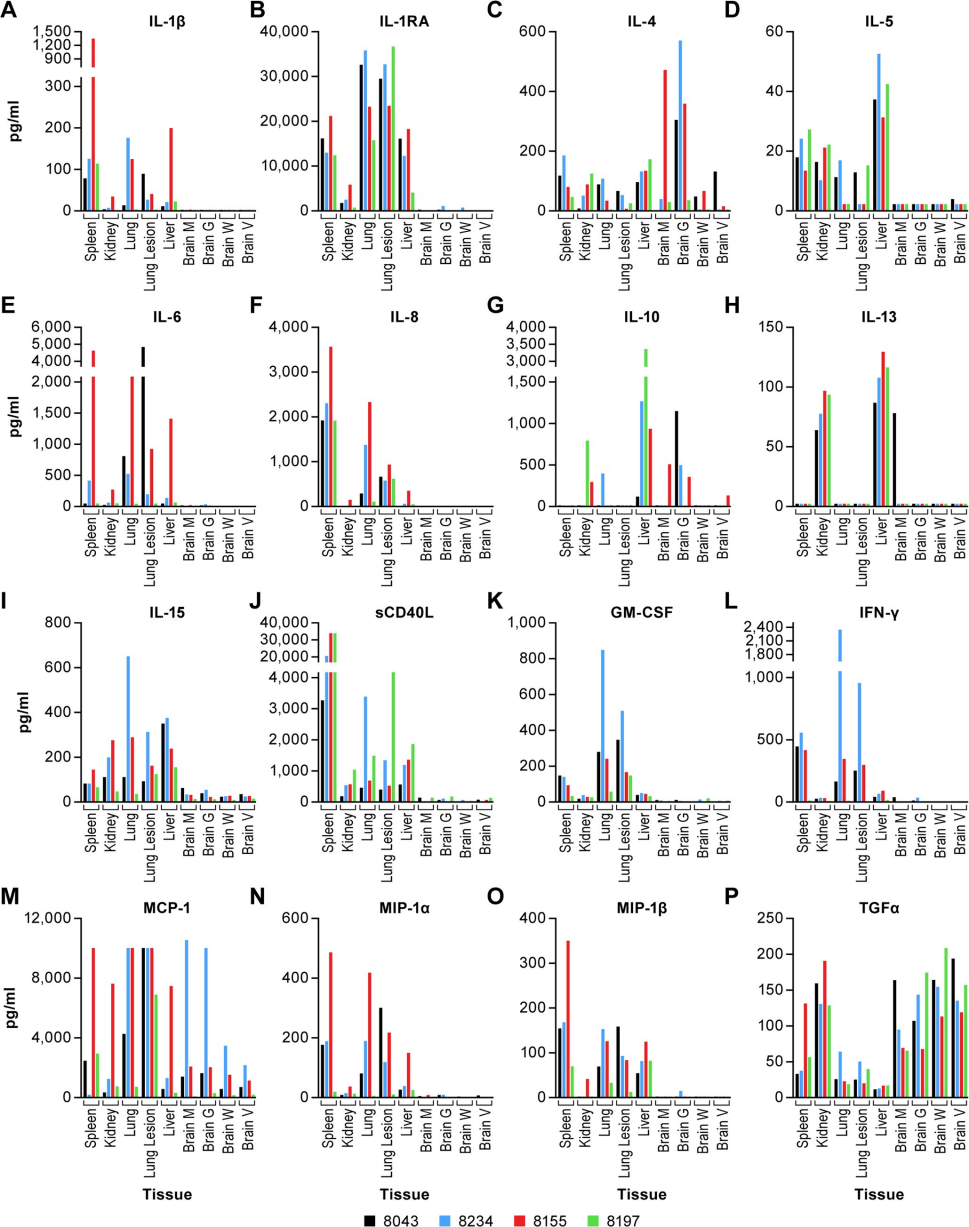
Cytokine analysis in tissues. A 23-plex bead-based cytokine assay was performed on tissues collected from four of the six animals included in this study. One animal is from the low dose group (8043-black) and the other three are from the high dose group. Samples from two animals were not tested due to tissue degradation. Tissues tested include the spleen, kidney, lung (both clear and tissue with apparent lesion), liver and four brain samples. Brain samples included a miscellaneous section (Brain M) and sections from the gray matter (Brain G), white matter (Brain W) and a section surrounding brain ventricles (Brain V) to include the ependyma and surrounding tissue.

Cytokines that appeared to be consistently upregulated in specific tissues relative to the two control animals include IL-4 in the brain; IL-5 in the liver; IL-8 in the lung; IL-10 in the liver; IL-13 in the kidney and liver; IL-15 in the kidney, lung and liver; IL-17A in the liver; GM-CSF in tissues other than the brain; IFN-γ in the spleen and lung; MCP-1 in most tissues; MIP-1α in the spleen and lung; MIP-1β in the spleen, lung and liver; and TGFα in the brain (Fig 6). Broadly evaluating the cytokine response in all tissues suggests that animal 8155 had a more robust response to infection than did other animals. Sampling of the surviving animal (8197) occurred several weeks after sampling of the other three animals and this animal was well past the acute phase of disease despite having focal brain lesions. With most analytes, the response measured in the survivor was not markedly different from other animals tested. There were some exceptions including IL-10 in the liver, sCD40L (CD154) in one lung sample and a decreased level of IL-15 and chemokines (MIP-1α, MIP-1β and MCP-1) relative to other animals. The reduced IL-15 and chemokine levels suggest decreased proliferation and recruitment of T cells and APCs in the tested tissues and would be consistent with convalescence.

In the brain, TGFα was expressed at high levels in all animals, including the convalescent animal. In animals that succumbed to NiV infection, only IL-4 was markedly different from the survivor, particularly in the gray matter (Fig 6). In one animal (8234), there was a much higher level of MCP-1 (CCL2) evident in the gray matter and in a brain tissue sample of undocumented origin.

In the lung of each animal, two regions of interest were sampled, one with no apparent lesion and one with a grossly apparent lesion, if evident. It should be noted that collection of “lesion” or “non-lesion” tissue is not precise and could include healthy and diseased tissue. In the surviving animal, the only cytokine that was elevated in the lung relative to other animals was CD40L, which is expressed on activated T cells and is suggestive of T cell infiltration into the lung. Among the acutely ill animals that succumbed to infection, there were no consistent findings that could point to a specific immune response that was a hallmark of NiV infection or disease progression. One animal (8155) appeared to have an elevated pro-inflammatory response with increases in IL-6, IL-8 and TNFα although this was noted in “non-lesion” tissue (Fig 6). A correlating response was not seen in tissue with an apparent lesion. One animal (8043) had elevated IL-6, MIP-1α and TNFα in “lesion” tissue also suggesting a local inflammatory response.

In the spleen, one animal (8155) had higher levels of several cytokines (IL-1β, IL-6, G-CSF, MIP-1α, MIP-1β, MCP-1, TGFα and TNFα) than other animals suggesting a high level of activity in this organ (Fig 6). There were no marked and consistent differences between animals in either the kidney or the liver except for elevated MCP-1 in the kidney of one animal.

### Tissue immune cell populations

These analyses were focused on tissues that were previously identified as target tissues for NiV infection, the lung and the brain, to determine if there were immune cell populations present that could be attributed to the disease. In these analyses, tissues were collected immediately following euthanasia from all three animals in the high dose group, including the survivor, and from only one animal in the low dose group due to tissue degradation in the other animals. Tissues were also collected from the two uninfected animals independent from the NiV exposure study. While the tissues analyzed by flow cytometry were similar to those used for cytokine analysis, they were different tissue sections.

**Fig 7:**
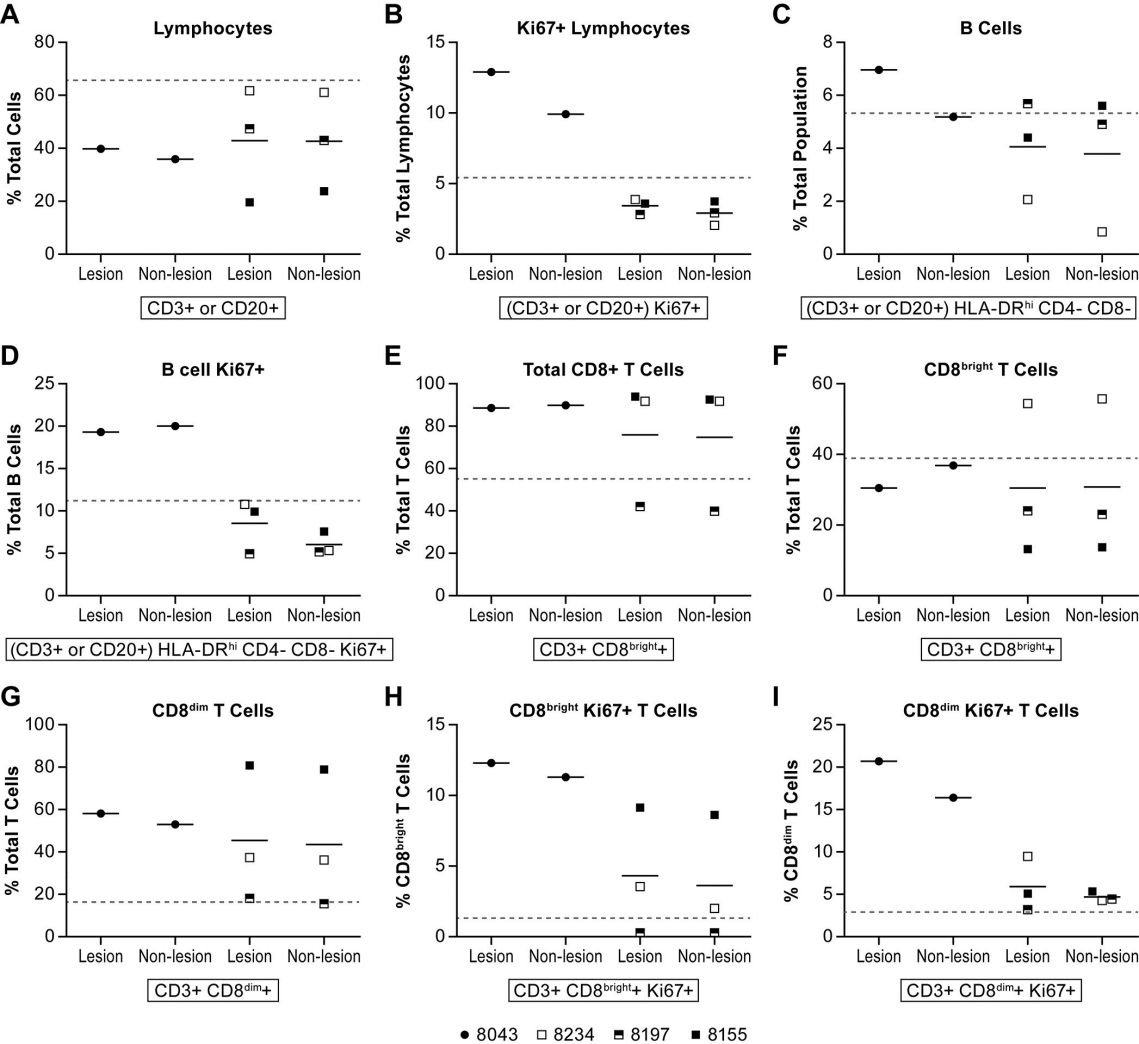
Lymphocyte cell populations in the lung. Flow cytometric analysis for quantification of lymphocytes collected from the lungs of four of six animals following exposure to NiV. Tissues were not collected from two animals due to tissue degradation. For each animal, samples from apparently unaffected tissue (non-lesion) and affected tissue (lesion) were collected. The specific markers used to distinguish lymphocytes are indicated below the individual panels. Analyses included the Ki67 marker to determine if cell populations were proliferating. Animals included in this analysis included 8043 (filled circle), 8234 (open square), 8197 (half filled square) and 8155 (filled square). Individual cell populations included (A) lymphocytes; (B) proliferating lymphocytes; (C) B cells; (D) proliferating B cells; (E) total CD8+ T cells; (F) CD8^bright^ T cells; (G) CD8^dim^ T cells; (H) proliferating CD8^bright^ T cells; and (I) proliferating CD8^dim^ T cells. Markers and antibody clones used for these analyses can be found in S3 Table. The dashed horizontal line indicates the mean value of equivalent cell populations collected from two control animals. The solid lines represent the mean for that dose group.

In the lungs, two different samples were collected, one with an apparent “lesion” and one without. As with tissues collected for cytokine analysis, while gross observation identified tissues as lesion or non-lesion, it should be appreciated that each tissue section could contain both normal and abnormal cell populations. In these non-perfused tissues, there were few distinct and consistent differences between tissues collected as “lesions” and “non-lesions”. In the two normal animals, approximately 65% of the cells analyzed were lymphocytes (Fig 7A), with approximately 5% of these cells expressing the Ki67 proliferation marker (Fig 7B). While the total number of lymphocytes in the lungs collected from infected animals was slightly lower than in uninfected animals, the percent of proliferating lymphocytes was consistent except for one animal where just over 10% of the total lymphocytes were Ki67+. In both normal and infected animals, less than 10% of the lymphocyte population consisted of B cells (Fig 7C). In one animal (#8043) about 20% of the B cells were Ki67+ suggesting B cell proliferation (Fig 7D). Of the CD3+ T cells identified in lungs of infected animals, over 85% were CD8+ T cells in all but the surviving animal, a number that was markedly higher than seen in uninfected animals (Fig 7E). In the surviving animal the total population of CD8+ T cells was approximately 40% in the two collected samples, a level slightly lower than the average of the two controls, but consistent with one of the control animals. In AGM, there are two populations of CD8+ T cells. CD8^bright^ and CD8^dim^, where the CD8^bright^ population has classical CD8 cytolytic activity and the CD8^dim^ cells have CD4 helper activity [30, 31]. In uninfected AGM, the CD8^bright^ population in the lung was about 30–50% of the total T cell population while the CD8^dim^ population was around 12–20%, depending upon the individual sample (Fig 7F and 7G). In one animal (#8043) both CD8^bright^ and CD8^dim^ populations had a high proportion of Ki67+ cells suggesting cell proliferation, while in one animal (#8155) only the CD8^bright^ population was proliferating (Fig 7H and 7I). In the convalescent animal, the proportion of Ki67+ CD8+ T cells in the lung was very low.

**Fig 8:**
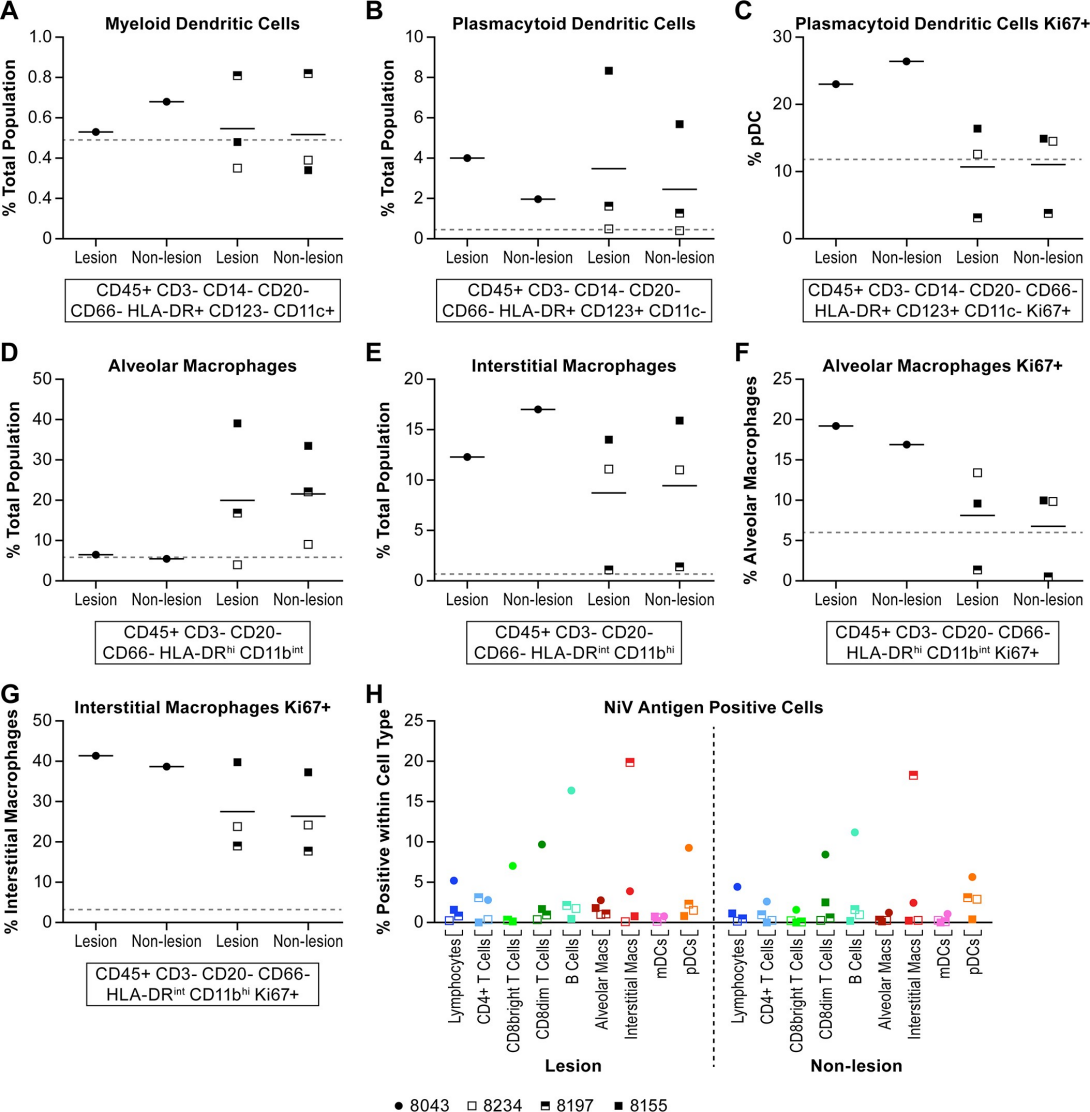
Antigen presenting cell populations in the lung. Flow cytometric analysis of populations of antigen presenting cells (APC) in the lungs of animals infected with NiV. Tissues were not collected from two animals due to tissue degradation. For each animal, samples from apparently unaffected tissue (non-lesion) and affected tissue (lesion) were collected. The specific markers used to distinguish APCs are indicated below the individual panels. Analyses included the Ki67 marker to determine if cell populations were proliferating. Animals included in this analysis included 8043 (filled circle), 8234 (open square), 8197 (half filled square) and 8155 (filled square). Individual cell populations included (A) myeloid dendritic cells (mDCs); (B) plasmacytoid dendritic cells (pDCs); (C) proliferating pDCs; (D) alveolar macrophages; (E) interstitial macrophages; (F) proliferating alveolar macrophages; and (G) proliferating interstitial macrophages. Cell populations isolated from the lung were also tested for the presence of NiV antigen (H). Markers and antibody clones used for these analyses can be found in S3 Table. The dashed horizontal line in panels A-G indicates the mean value of equivalent cell populations collected from two control animals. The solid lines in panels A-G represent the mean for that dose group.

Evaluation of APCs in the lungs found a very small (<1.0%) total CD45+ cell population were myeloid dendritic cells (DC) (mDC) in both infected and uninfected animals (Fig 8A), while the plasmacytoid (pDC) population was up to 8% in one acutely ill animal compared to less than 1.5% in the uninfected animals (Fig 8B). Evaluation of APC proliferation found that 10–25% of the pDC from acutely ill animals were Ki67+ while <5% were Ki67+ in the convalescent animal (Fig 8C). In uninfected animals, the number of Ki67+ pDCs was 4–25%. Populations of macrophages were defined as alveolar and interstitial and were differentiated by the expression of HLA-DR and CD11b where alveolar macrophages were HLA-DR^hi^ CD11b^int^ and interstitial macrophages as HLA-DR^int^ and CD11b^hi^. In two of the infected animals, alveolar macrophages were the predominant population and in one of the acutely ill animals, the interstitial population was predominant (Fig 8D and 8E). In one animal (8155), both alveolar and interstitial macrophages were elevated when compared to uninfected animals (Fig 8D and 8E). This finding is consistent with the elevated levels of MIP-1α (Fig 6) also found in the lung tissue of this animal. Evaluation of Ki67 expression in both alveolar and interstitial macrophages indicated that alveolar macrophages were proliferating in all animals except the survivor and interstitial macrophages were proliferating in all animals with the survivor having the lowest frequency of proliferating cells (Fig 8F and 8G). In uninfected animals, alveolar macrophages represented 2–8% of the population while interstitial macrophages were <2% of the population.

In addition to cell surface markers, individual populations were also stained for NiV antigen. One of the acutely ill animals (#8043) had higher levels of NiV antigen in B cells, CD4+ and CD8+ T cells, pDCs and mDCs, than the other animals (Fig 8H). In the convalescent animal, NiV antigen was very evident in interstitial macrophages with some antigen found in CD4+ T cells and pDCs.

### BAL cell populations

To determine cell populations of the exudate in the lungs, bronchoalveolar lavage (BAL) was performed immediately before euthanasia of the four animals that were euthanized. Samples were not attainable from two animals that succumbed to the infection. BAL was not performed over the course of disease as there was concern about altering the disease course or potentially inducing artifactual inflammation. Data from the two control animals is included for comparison.

**Fig 9:**
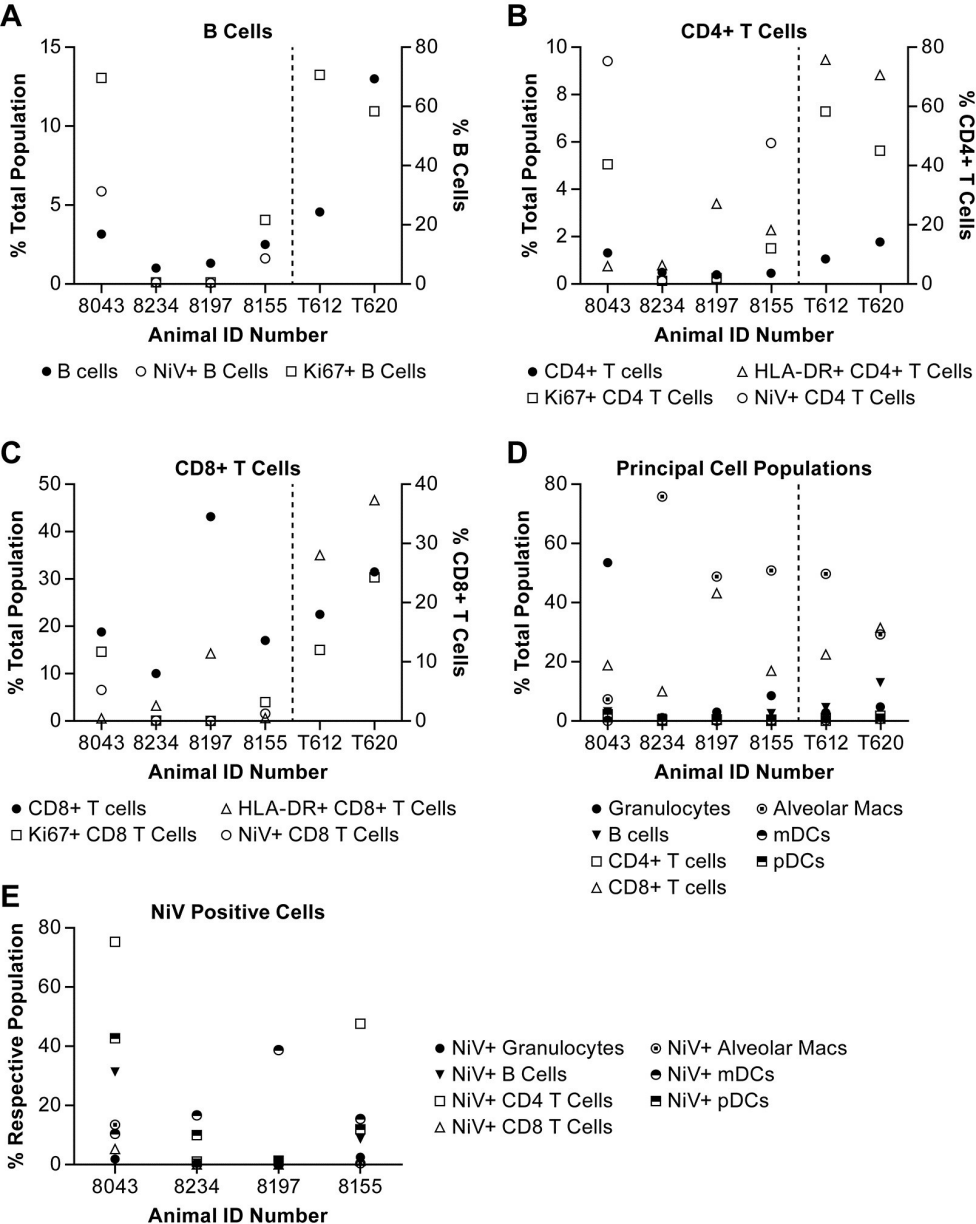
Immune cell populations in bronchoalveolar lavage. Flow cytometric analysis of BAL collected from animals immediately prior to necropsy. Samples were collected from four of six animals included in this study. Also included are data from two uninfected control animals (T612 and T620). Analysis focused on seven principal cell populations that were expected in the BAL and included the proliferation marker Ki67 and determination of the presence of NiV antigen using a virus glycoprotein (G) specific polyclonal antiserum [25]. Adaptive immune response cell populations evaluated included (A) B cells; (B) CD4+ T cells and (C) CD8+ T cells. The distribution of all cell populations evaluated in individual animals is provided in (D) and (E) shows which cell populations were NiV antigen positive.

Overall B cell populations in the BALs were reduced relative to control animals. In addition, the percentage of B cells that were proliferating (Ki67+) in the two control animals was relatively high (~60%) whereas only one infected animal (8043) had a similarly high level of B cell proliferation (Fig 9A). Two of the animals had negligible Ki67+ B cells at euthanasia and the fourth animal (8155) was around 20%. The two animals with higher levels of proliferating B cells also had a slightly higher level of total B cells, both of which were lower than the two control animals. The two animals with the highest percentage of Ki67+ B cells also had about 5% of the total population NiV antigen positive.

Total populations of CD4+ T cells in the BAL from infected animals were similar with uninfected animals (Fig 9B), but levels of proliferating (Ki67+) or activated (HLA-DR+) CD4+ T cells were considerably lower in all of the infected animals than was measured in the two control animals. The two animals that had evidence of NiV antigen in the B cells were also NiV antigen positive in their CD4+ T cells.

The total population of CD8+ T cells in the BAL from three of the four NiV infected animals was lower than the uninfected controls, but one animal, the survivor, had a markedly elevated CD8+ T cell level compared to the other animals (Fig 9C). Even though the CD8+ T cell level was high, there was little evidence of either proliferating or activated CD8+ T cells in this animal. There was also a very small population of NiV antigen-positive CD8+ T cells detected in only two of the animals. These data suggest a role for CD8+ T cells in convalescence, but a limited response in the acutely ill animals.

The increased population of immune cells in the BAL was not necessarily reflective of the level of pulmonary disease based on total loss of lung volume. Of the animals sampled, one (8234) had the highest measure of hyperdensity in the lungs reflecting consolidation/infiltrates with secondary loss of lung volume. This animal had the lowest levels of lymphocytes and granulocytes of the animals sampled. This animal also had low levels of myeloid and plasmacytoid DCs, but higher levels of alveolar macrophages (Fig 9D). These data suggest the cellular component of the exudate in the lungs consists primarily of alveolar macrophages. Evaluation of BAL cell populations found NiV antigen primarily in APCs, but two animals were also NiV antigen positive in B and T cells (Fig 9E). The only population of cells that was NiV antigen positive in the survivor was myeloid DCs.

### Brain cell populations

**Fig 10:**
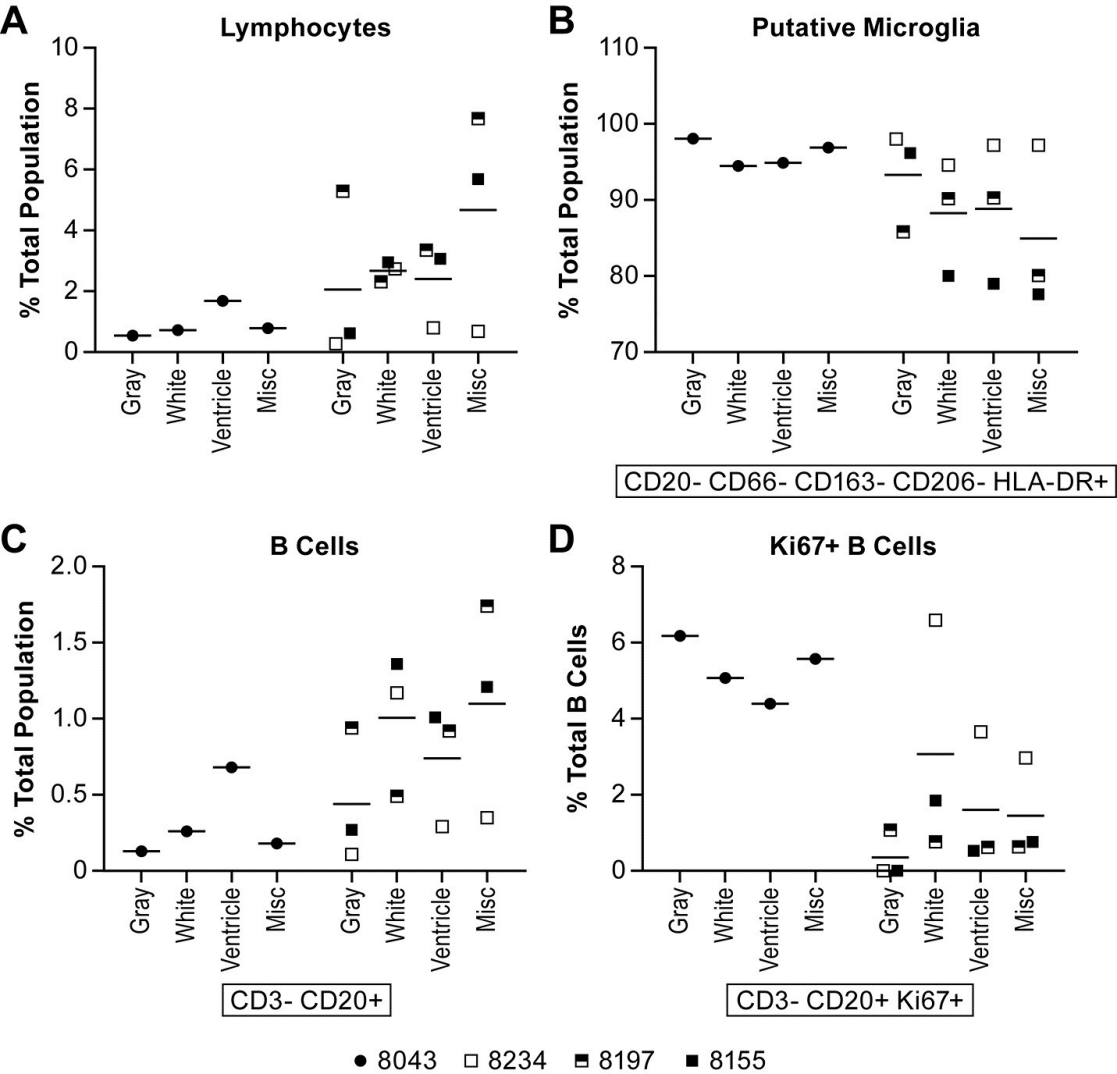
Lymphocyte, microglia and B cell populations in the brain. Flow cytometric analysis of cell populations in brain tissue collected at necropsy. Tissues were not collected from two animals due to tissue degradation. For each animal, samples were collected from three different regions of the brain, the gray matter, white matter and tissue immediately adjacent to the ventricles to include the ependyma. A random tissue section was also collected (Misc) if it appeared on gross examination to be of interest. The specific markers used to distinguish cell populations are indicated below the individual panels. Analyses included the Ki67 marker in B cells to determine if cell populations were proliferating. Animals included in this analysis included 8043 (filled circle), 8234 (open square), 8197 (half filled square) and 8155 (filled square). Individual cell populations included (A) lymphocytes; (B) putative microglia; (C) B cells; (D) Ki67+ B cells. Markers and antibody clones used for these analyses can be found in S3 Table. The solid lines represent the mean for that dose group.

**Fig 11:**
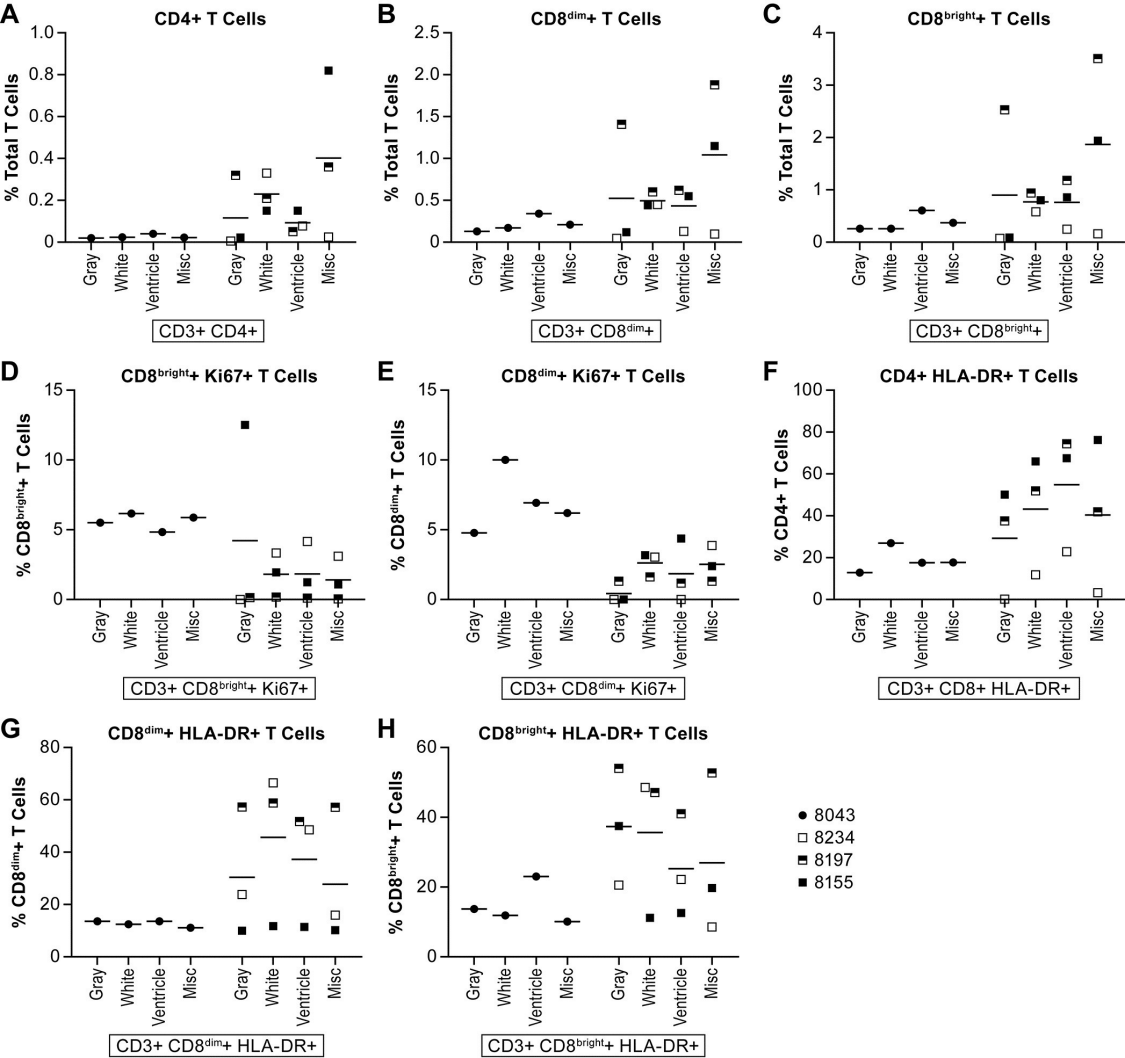
T cell populations in the brain. Tissues were collected as described in the legend for Fig 10. The specific markers used to distinguish cell populations are indicated below the individual panels. Analyses included the Ki67 marker to determine if cell populations were proliferating and the HLA-DR to determine if cells were activated. Animals included in this analysis included 8043 (filled circle), 8234 (open square), 8197 (half filled square) and 8155 (filled square). These analyses included (A)CD4+ T cells; (B) CD8^dim^ T cells; (C) CD8^bright^ T cells; (D) proliferating CD8^bright^ T cells; (E) proliferating CD8^dim^ T cells; (F) HLA-DR+ CD4+ T cells; (G) HLA-DR+ CD8^dim^ T cells; (H) HLA-DR+ CD8^bright^ T cells. The solid lines represent the mean for that dose group.

**Fig 12:**
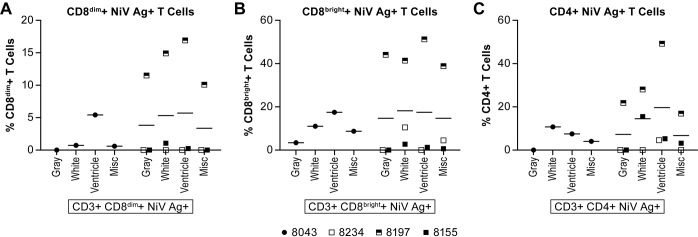
Nipah virus antigen positive T cells in the brain. Tissues were collected as described in the legend for Fig 10. Animals included in this analysis included 8043 (filled circle), 8234 (open square), 8197 (half filled square) and 8155 (filled square). Detection of intracellular antigen was achieved using a NiV GP reactive polyclonal antisera generated in rabbits. Analyses included (A) NiV antigen+ CD8^dim^ T cells; (B) NiV antigen+ CD8^bright^ T cells; and (C) NiV antigen+ CD4+ T cells. The solid lines represent the mean for that dose group.

As expected, the total lymphocyte population in the brain was very low in two of the animals, one in each group, but in the other two animals, including the survivor, the lymphocytes were up to 8% of the total population depending on the part of the brain sampled (Fig 10A). The majority of the cells isolated were identified as microglia (Fig 10B), although this population has not been clearly defined in the AGM. Of the lymphocytes samples, B cells made up <2% of the total population and T cells <5% in most of the animals (Figs 10C and 11A–11C). The surviving animal had >4% CD8+ T cells within the total CD3+ T cell population in two of the brain tissues sampled (Fig 11B and 11C), the majority of which were CD8^bright^+ T cells suggesting cytolytic activity (Fig 11C). In two of the acutely ill animals, 4–6% of the B cells were proliferating (Ki67+) (Fig 10D). A higher percentage of both CD8^bright^+ and CD8^dim^+ T cells were also found to be proliferating in this animal (Fig 11D and 11E). Evaluation of T cell activation using the HLA-DR marker found that many of the CD4+ and CD8+ T cell populations isolated from the brain of each of the animals had a high proportion (>30%) of HLA-DR+ cells (Fig 11G and 11H). Staining for NiV antigen was positive in a negligible population of CD8^dim^+ T cells in all animals except the survivor where >10% of the population was NiV antigen positive in the four samples collected (Fig 12A). Similarly, over 40% of the CD8^bright^+ and over 20% of CD4+ T cells were NiV antigen positive in all four brain samples collected from the survivor (Fig 12B and 12C).

## Discussion

The primary objective of this study was to use an intermediate particle size aerosol exposure to determine if NiV deposition in the upper respiratory tract [27] would lead to disease that mimics that seen in humans, including development of neurological disease. An additional objective was to characterize the immune response to NiV infection to gain a broader understanding of pathogenicity in the AGM model that might be translatable to human disease. The Malaysian isolate of NiV was used in these studies to retain consistency with the initially described AGM model for NiV infection and with our previously described work with IT inoculation and small-particle aerosol exposure [22]. There is some anecdotal evidence suggesting that the Bangladesh isolate of NiV may be more closely aligned to development of respiratory disease in humans while the Malaysian isolate has been associated with neurological disease. However, in this study and in our previous work, respiratory disease has been more predominant following NiV infection with no overt evidence of neurological disease outside that identified by MRI.

The studies described here found that exposure to the intermediate particle aerosol led to an extended disease course that did not have the prominent respiratory features and rapidly progressing disease that we, and others, had identified using IT or small-particle aerosol exposure [21, 22, 28, 29]. While respiratory disease was still a critical component of the disease process, examination by CT found far less edema and consolidation than previously described. Although respiratory disease was evident in these animals, overt neurological disease was not. In fact, only the use of MRI allowed identification of clearly distinct lesions in the brains of three animals, including the lone survivor. Many of those lesions were similar to reported abnormalities in patients infected with NiV. Some of the lesions showed restricted diffusion consistent with small acute/subacute infarcts, while a few showed increased signal only on FLAIR and T2-weighted images without restricted diffusion. Very similar findings have been described in the infected patient populations in Singapore and Malaysia [8, 10, 11, 32, 33] which described brain lesions with or without restricted diffusion. We believe the small lesions with restricted diffusion reflect small ischemic foci related to vasculitis and thrombosis. Those findings are supported by the identification of viral antigen in endothelial cells in the brain (Fig 4C) and the presence of what appears to be a resolving microthrombus in one of the identified lesions (Fig 4E and 4F). Also, many of the lesions in Nipah survivors resolved, which is analogous to what we saw in the lone survivor in our group; lesions that were seen on day 16 and day 22, had resolved by day 36. In patients, dystrophic calcifications were eventually seen as bright lesions on T1 weighted images and foci of decreased signal on SWI images [11]. We did not see similar changes in our animals, probably because they did not survive long enough for calcifications to develop. While we were unable to sample all the identified lesions in these animals, these data suggest that changes in the brain were due to vascular changes rather than virus induced infiltration of immune cells leading to classical histological encephalitis. These data, in addition to the limited infection of neurons, further support the hypothesis that NiV is not a neurotropic virus and that clinical determination of encephalitis is the result of non-inflammatory neurological changes.

To evaluate local immune responses to viral infection, and potentially identify hallmarks of the disease, spleen, kidney, liver, brain, lung and BAL samples were collected at study termination. Data from these samples were compared to historical control samples collected from two uninfected animals. Evaluation of the cytokine response in the lungs suggests a local immune response to NiV infection. The low levels of IL-1β, IL-6 and IL-8 levels in the lungs of some of the animals are suggestive of a limited inflammatory response at the terminal phase of disease, while the high levels of IL-1RA suggest a robust effort to limit an inflammatory response. Expression of IL-6 and IL-8 are regulated by NF-κB, which can be induced by IL-1β [34–36]. The high levels of IL-1RA would mitigate this effect by binding to and blocking the IL-1 receptor. It is possible that local levels of proinflammatory cytokines were elevated earlier in the disease course and the status at necropsy represents successful regulation of the proinflammatory response. It is also possible that NiV can inhibit an NF-κB driven proinflammatory response. The related mumps virus uses the SH protein to block NF-κB activation [37], but SH is a protein that is not present in NiV. Measles virus, however, has been shown to activate NF-κB [38] demonstrating that paramyxoviruses use different approaches for regulating host cell immunity suggesting that NiV may regulate NF-κB activity through a yet undefined mechanism.

The relatively high level of chemokines in the lungs of the acutely ill animals suggests recruitment of immune cell populations such as monocytes, T cells and dendritic cells. Previous work evaluating chemokine levels in NiV infected airway epithelial cells found that these cells released high levels of MCP-1, particularly from small airway epithelial cells [39]. Consistent with Escaffre et al., MCP-1 levels were very high in the lung samples from NiV infected AGM. The elevated CD8+ T cell population correlates with the high levels of MCP-1 found in the lung samples with CD8+ T cell populations higher in animals that succumbed in the acute phase and lower in the survivor. The majority of the CD8+ T cell population in the lung samples from two animals consisted of the CD8^dim^+ subtype suggesting T cell help rather than the cytolytic activity associated with the CD8^bright^ population. The CD8^dim^+ T cell levels in the lungs from all three of the acutely ill animals were elevated compared to the naïve controls, while levels in the survivor were approximately the same. Similarly, CD8+ T cells were the predominant lymphocyte population in BAL, but here the survivor had a higher percentage of CD8+ T cells than did those animals that succumbed during the acute phase, suggesting that some of the CD8+ T cells may have been migrating from the tissue into the interstitial space. Cytokine analysis of the BAL was not performed.

The elevated chemokine levels in the lung tissue also suggest recruitment of APCs. The elevated level of pDCs, alveolar macrophages and interstitial macrophages in the lungs of most animals also suggests recruitment to the tissues. The populations of APCs in the BAL were not markedly different from the control animals, however, one animal had over 50% of the isolated immune cell population from its BAL composed of granulocytes. Specific delineation of the type of granulocytes was not performed, but they are most likely neutrophils and basophils. While the presence of these cells may suggest a secondary bacterial infection in the lung, there was no histological evidence that this was the case.

Evaluation of cell populations in the brain identified microglia as the predominant non-neuronal cell populations in both the naïve and NiV infected animals. As expected, the proportion of lymphocytes was very low in most samples tested. Of the lymphocytes identified, they were predominantly CD8+ T cells. In general, the animals that succumbed to NiV infection had lower proportions of T cells in their brain tissue compared to the surviving animal. Interestingly, a large proportion of the T cells isolated from the brain of the survivor were also NiV antigen positive, particularly the CD8+ T cells. While there is currently no evidence that NiV can infect and replicate within T cells, these data are suggestive and may represent a potential reservoir for virus latency or persistent infection.

In summary, the data presented here demonstrate that NiV infection in AGMs can mimic human disease given an appropriate exposure route and viral dose. While these findings are helpful in understanding NiV induced disease, the infection in this model is still more lethal than reported in humans. Subsequently, further work is required to fully understand the critical components of disease progression and to identify specific correlates of disease that may allow identification of successful treatment options for NiV infection. In addition, studies carefully evaluating infection of AGM with the Bangladesh isolate of NiV will be important for determining if there is a disease phenotype specific to the Bangladesh virus variant as has been suggested based on clinical outcome data.

## Supporting information

S1 Fig Cytokine analysis from tissues of uninfected control animals. Cytokine expression in tissues collected from two uninfected AGM using a 23-plex bead-based assay. Tissues analyzed include plasma, spleen, kidney, left and right lung lobes, tracheobronchial lymph node, liver and spleen.
(TIF)

S1 Table Back titration and physical characteristics of aerosol exposure. Characteristics of the aerosol exposure for each individual animal and back-titration of the biosampler integrated into the aerosol chamber. Aerosol parameters included the minute volume, mean particle size, geometric standard deviation (GSD) for each particle size (1.2 is optimal), the total number of particles in the exposure and the viable virus titer. Animals were exposed on two different days to accommodate the imaging schedule.
(DOCX)

S2 Table Descriptive findings animals included in the study. (A) Radiographic findings in the lungs from each individual animal over the course of the study. (B) Gross pathology descriptions of select tissues from each individual animal at the time of necropsy. (C) Histological findings from each individual animal in select organs.
(DOCX)

S3 Table Antibody panel used for flow cytometry study in the lung, brain and BAL. Table provides the target marker, antibody clone used and the fluorophore conjugated to each individual antibody. The polyclonal antisera used for detecting NiV G protein was developed in rabbit inoculated with purified NiV G protein.
(DOCX)
