# Supplementary figures and images for: Aerosol exposure to intermediate size Nipah virus particles induces neurological disease in African green monkeys
Source: PLoS Negl Trop Dis. 2018 Nov 21;12(11):e0006978. doi: 10.1371/journal.pntd.0006978 (PMC6281276; doi:10.1371/journal.pntd.0006978)

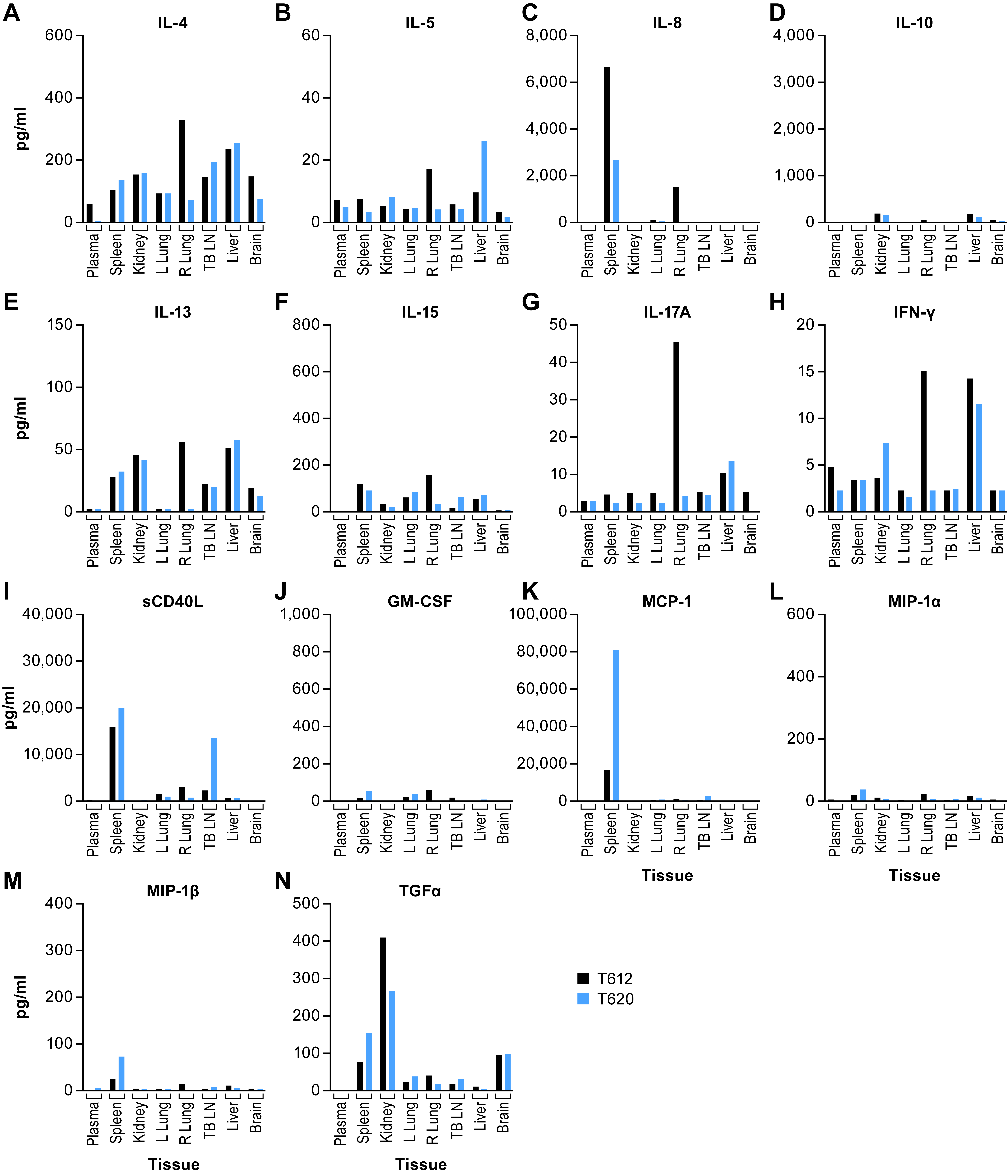

Supplement: S1 Fig — Cytokine expression in tissues collected from two uninfected AGM using a 23-plex bead-based assay. Tissues analyzed include plasma, spleen, kidney, left and right lung lobes, tracheobronchial lymph node, liver and spleen. (TIF) [file pntd.0006978.s001.tif]
