# Supplementary material for: Aerosol exposure to intermediate size Nipah virus particles induces neurological disease in African green monkeys
Source: PLoS Negl Trop Dis. 2018 Nov 21;12(11):e0006978. doi: 10.1371/journal.pntd.0006978 (PMC6281276; doi:10.1371/journal.pntd.0006978)
Supplement: S1 Table — Characteristics of the aerosol exposure for each individual animal and back-titration of the biosampler integrated into the aerosol chamber. Aerosol parameters included the minute volume, mean particle size, geometric standard deviation (GSD) for each particle size (1.2 is optimal), the total number of particles in the exposure and the viable virus titer. Animals were exposed on two different days to accommodate the imaging schedule. (DOCX) [file pntd.0006978.s002.docx]

**Supplemental Table 1**

| **Animal ID** | **Min. Vol (L/min)** | **Particle Size (µm)** | **GSD** | **Particle Counts (log_10_)** | **Exposure Dose (pfu)*** |
| --- | --- | --- | --- | --- | --- |
| 08043 | 0.610 | 7.19 | 1.36 | 5.08 | 22 |
| 08164 | 1.100 | 7.18 | 1.37 | 5.04 | 79 |
| 08150 | 0.456 | 7.10 | 1.40 | 5.11 | 90 |
| 08234 | 0.564 | 6.52 | 1.47 | 5.34 | 1197 |
| 08197 | 0.875 | 6.62 | 1.46 | 5.32 | 408 |
| 08155 | 0.260 | 6.10 | 1.53 | 5.18 | 499 |

*Calculated based on titration of material collected on aerosol sampling filter
