# Supplementary material for: Aerosol exposure to intermediate size Nipah virus particles induces neurological disease in African green monkeys
Source: PLoS Negl Trop Dis. 2018 Nov 21;12(11):e0006978. doi: 10.1371/journal.pntd.0006978 (PMC6281276; doi:10.1371/journal.pntd.0006978)
Supplement: S2 Table — (A) Radiographic findings in the lungs from each individual animal over the course of the study. (B) Gross pathology descriptions of select tissues from each individual animal at the time of necropsy. (C) Histological findings from each individual animal in select organs. (DOCX) [file pntd.0006978.s003.docx]

**Supplemental Table 2A. Radiographic findings in lungs of African green monkeys inoculated with Nipah virus between 2 and 36 days post infection.** Imaging and clinical observation were made every two days until the animal met euthanasia criteria.

| **Animal** | **Group** | **Day 2** | **Day 4** | **Day 6** | **Day 8** | **Day 10** | **Day 14** | **Days 22-36** |
| --- | --- | --- | --- | --- | --- | --- | --- | --- |
| 08043 | Low | Lungs normal. No significant infiltrates. | Lungs normal. No significant infiltrates. | Lungs normal. No significant infiltrates. | Lungs normal. No significant infiltrates. | Lungs normal. No significant infiltrates. | NA | NA |
| 08164 | Low | Lungs consistent with baseline. | Lungs consistent with baseline. | Lungs consistent with baseline. | Opacity noted in baseline in upper right lobe more pronounced; air bronchogram noted in right lower lobe | Ground glass infiltrate noted in right and left upper lobe and right lung adjacent to the heart | NA | NA |
| 08150 | Low | Lungs consistent with baseline. | Subtle opacity in left upper lobe; otherwise lungs consistent with baseline. | Lungs consistent with baseline. Opacity resolved | Ground glass opacity, upper right lobe; Dense consolidation lower left lobe with ground glass opacity and air bronchogram; ground glass opacity right upper lobe | Infiltrate in lower left lobe is enlarged; area of infiltrate in lower left lobe has enlarged; focal consolidation in right upper lobe; thymic atrophy; hilar adenopathy | NA | NA |
| 08234 | High | Small opacity in upper left lobe; Subtle ground glass opacity in right lung base | Upper lobe opacity is larger and more dense; right lung opacity has resolved | Right lung opacity has returned, but subtle. | Left upper lobe opacity has returned and is larger; Opacity on right upper lobe; Right lower lobe shows bronchovascular thickening | Focal consolidation in upper left lobe; ground glass opacity in right upper and lower lobes; Nodular opacity in left lower lobe | NA | NA |
| 08197 | High | Small opacity in upper right lobe | Upper right lobe opacity resolved; multifocal peribronchial opacity in lower right lobe, some alveolar involvement | Previous opacities largely resolved; large dense infiltrate in upper right lobe with air bronchogram; pretracheal LN enlarged | Infiltrate in upper right lobe smaller but still substantial; LN further enlarged; all else resolved | Noted abnormalities resolving | Noted abnormalities largely resolved | Multifocal ground glass opacity developed in middle right lobe (d 22) and resolved |
| 08155 | High | Ground glass infiltrate in left and right upper lobes, left lower lobe | Previously noted abnormalities have resolved | Ground glass opacities in left upper lobe | Largely unchanged from previous | Left upper lobe opacities resolved; ground glass opacity in left lower lobe | NA | NA |

LN: Lymph node

**Supplemental Table 2B: Gross Pathologic Assessment at Necropsy**

| **Animal** | **Group** | **Term Day** | **Lung Lobes** | **Peripheral Lymph Nodes^1^** | **Bowel** | **Spleen** | **Heart/Brain** | **Other** |
| --- | --- | --- | --- | --- | --- | --- | --- | --- |
| 08043 | Low | 13 | Mildly congested; multifocal areas of hemorrhage; significant congestion in right caudodorsal lobes | A&I Minimally congested. TB slightly edematous | Minimal liquid ingesta and digesta; moderate gas | Turgid; slightly friable | Unremarkable | Mild edema in mediastinum and pericardial connective tissue |
| 08164 | Low | 13 | All lobes dark red; diffuse, mild interstitial pneumonia | Variable edema and probable congestion | Moderately distended with gas | 3x enlarged by congestion | Unremarkable | Delayed necropsy; carcass showed mild autolysis; liver moderately mottled, friable; moderate transudate in thoracic cavity; petechiae on mucosa of urinary bladder |
| 08150 | Low | 10 | All lobes with congestion and hemorrhage; interstitial pneumonia, diffuse, mild | Unremarkable | Focal, firm fecal material at colon-rectum junction; severely distended by gas | Unremarkable | Unremarkable | Delayed necropsy; carcass showed mild to moderate cytolysis; marked and multifocal petechiae on mucosa of urinary bladder |
| 08234 | High | 10 | Caudodorsal lobes multifocally congested and only partially collapse | A&I Minimally congested; TB are congested and slightly edematous | Minimal amounts of ingesta and digesta; abundant gas | Turgid, slightly friable | Unremarkable | Minor petechial hemorrhage on shoulders; subcutaneous hemorrhage at base of tongue; mucosa of urinary bladder congested |
| 08197 | High | 36 | Mildly congested along dorsal surface | Unremarkable | Unremarkable | Unremarkable | Unremarkable | Multi-focal petechial rash in areas clipped or abraded |
| 08155 | High | 11 | Mildly congested with focally extensive area of dark red consolidation in the right craniodorsal lobe | A&I slightly congested; TB slightly enlarged, edematous | Minimal amounts of ingesta and digesta; minimal gas | Unremarkable | Unremarkable | Minimal edema of mediastinum and pericardial connective tissue; Multi-focal petechial rash in areas clipped; red tinged foamy material draining from nostrils |

^1^Axillary, inguinal and tracheobronchial lymph nodes.

**Supplemental Table 2C: Histological assessment of collected tissues**

| **Animal** | **Group** | **Lung** | **Lymph Nodes** | **Spleen** | | | **Liver** | | **Kidney** | **Brain** | **Pancreas** | |
| --- | --- | --- | --- | --- | --- | --- | --- | --- | --- | --- | --- | --- |
| 08043 | Low | Diffuse, mild alveolar congestion with necrotizing vasculitis, hemorrhage and edema. NiV antigen in histiocytes, syncytial cells, endothelial cells and pneumocytes | Germinal center degeneration, edema, hemorrhage. NiV antigen in histiocytes and endothelial cells. | | White pulp necrosis, degeneration with hemorrhage. Vasculitis, syncytia; NiV antigen in histiocytes and endothelial cells | Diffuse, mild vacuolar degeneration | | Multi-focal glomerular and interstitial congestion with edema. NiV antigen in histiocytes and endothelial cells | | No significant lesions. Rare NiV antigen staining of neurons or endothelium | Multifocal degeneration and necrosis of islets with acinar degeneration and hemorrhage. NiV antigen in histiocytes, syncytial cells and endothelial cells. |  |
| 08164 | Low | Diffuse peribronshiolar congestion with alveolar degeneration, lymphohistiocytic infiltrates and edema. NiV antigen in few histiocytes and endothelial cells | Multifocal germinal center depletion with edema and hemorrhage. NiV antigen in histiocytes. | | Multifocal white pulp degeneration with hyaline, hemorrhage, reactive endothelium and multifocal syncytia. NiV in histiocytes, rare endothelial cells | Multifocal sinusoidal congestion with vasculitis and minimal hepatocellular degeneration. NiV antigen in rare histiocytes and endothelium. | | Diffuse glomerular and interstitial congestion with edema. NiV antigen in few endothelial cells of small vessels and smooth muscle of larger arteries. | | No significant lesions in cerebrum or cerebellum. Rare NiV antigen staining of neurons or endothelium Meningitis with hemorrhage in brainstem with some antigen positivity in endothelium. | Multifocal degeneration of islets with acinar degeneration, lymphocytic infiltrates and syncytia. |  |
| 08150 | Low | Diffuse fibrinonecro-hemorrhagic bronchointerstitial pneumonia with vasculitis, edema, fibrin thrombi and syncytia. NiV antigen multifocally in histiocytes, endothelial cells and pneumocytes | Focally extensive lymphoid necrosis with depletion, vasculitis, hemorrhage and edema. NiV antigen in endothelial cells and few histiocytes. | | White pulp degeneration with hemorrhage, fibrin thrombi, vasculitis and syncytia. NiV antigen in endothelial cells and histiocytes. | Multifocal and random hepatocellular degeneration and necrosis with vasculitis, lymphocytic inflammation and edema. NiV antigen in few histiocytes and endothelial cells. | | Mild, multifocal, lymphocytic vasculitis with congestion and edema; few syncytia. NiV antigen in endothelial cells and smooth muscle cells of one vessel | | No significant lesions. NiV antigen in a few histiocytes and endothelial cells | Multifocal degeneration of islets with acinar degeneration and lymphocytic infiltrates. NiV antigen in areas of degeneration and necrosis as well as islets |  |
| **Animal** | **Group** | **Lung** | **Lymph Nodes** | | **Spleen** | **Liver** | | **Kidney** | | **Brain** | **Pancreas** |  |
| 08234 | High | Multifocal peribronchiolar congestion with minimal alveoalar degeneration; lymphohistiocytic infiltrates with edema and fibrin. NiV antigen in rare histiocytes and endothelial cells | Minimal multifocal germinal center depletion with edema, draining hemorrhage, histiocytes. No significant NiV antigen. | | Multifocal white pulp degeneration with hemorrhage; reactive endothelium, fibrin and reactive endothelial cells. NiV antigen in a few histiocytes | Random multifocal moderate to severe hepatocellular degeneration; Mallory bodies. No significant NiV antigen. | | Multifocal glomerular and interstitial congestion with edema and tubular dilation with protein. No significant NiV antigen. | | No significant lesions. No NiV antigen. | No significant lesions. No NiV antigen. |  |
| 08197 | High | Minimal multifocal alveolar congestion. NiV antigen in a few histiocytes. | Mild edema, draining hemorrhage and sinus histiocytes. NiV antigen in few histiocytes in mesenteric LN. | | Multifocal white pulp regeneration with reactive endothelium and red pulp edema. NiV antigen in a few histiocytes. | Minimal periportal hepatocellular vacuolar degeneration. No significant NiV antigen. | | Incidental, focal lymphohistiocytic nephritis. No significant NiV antigen. | | No significant lesions except that identified by MR. No significant NiV antigen. | No significant lesions. No significant NiV antigen. |  |
| 08155 | High | Diffuse, mild interstitial and alveolar congestion with perivascular hemorrhage, edema, fibrin and a proteinaceous alveolar exudate. Focal alveolar degeneration with hemorrhage. Minimal NiV antigen in histiocytes. | Diffuse germinal center depletion with edema, draining hemorrhage and sinus histiocytes. NiV antigen present in medullary histiocytes. | | Multifocal white pulp degeneration with hemorrhage, reactive endothelial cells, fibrin and multifocal syncytia. NiV antigen in a few histiocytes. | Focally extensive sinusoidal congestion with mild, diffuse hepatocellular vacuolar degeneration. No significant NiV antigen. | | Multifocal glomerular and interstitial congestion with dilated tubules and edema. | | Minimal focal lymphohistiocytic infiltrates in the brainstem. No significant NiV antigen. | Minimal multifocal congestion. NiV antigen in a focal cluster of cells. |  |
