# Supplementary material for: Aerosol exposure to intermediate size Nipah virus particles induces neurological disease in African green monkeys
Source: PLoS Negl Trop Dis. 2018 Nov 21;12(11):e0006978. doi: 10.1371/journal.pntd.0006978 (PMC6281276; doi:10.1371/journal.pntd.0006978)
Supplement: S3 Table — Table provides the target marker, antibody clone used and the fluorophore conjugated to each individual antibody. The polyclonal antisera used for detecting NiV G protein was developed in rabbit inoculated with purified NiV G protein. (DOCX) [file pntd.0006978.s004.docx]

**Supplemental Table 3-Antibody panel used for flow cytometric analysis of tissues and BAL**

**Tissue and BAL Panel**

| Antigen | Clone | Fluorophore |
| --- | --- | --- |
| CD66abce | TET2 | FITC |
| CD14 | M5E2 | Brilliant Violet 570 |
| CD11c | S-HCL-3 | APC |
| CD45 | D058-1283 | APC-R700 |
| CD16 | 3G8 | Brilliant Violet 785 |
| Anti-NiV G protein |  | PE |
| CD8α | SK1 | PE/Dazzle594 |
| CD206 | 19.2 | PE-Cy5 |
| CD123 | 7G3 | PE-Cy7 |
| HLA-DR | L243 | APC/Fire 750 |
| CD11b | ICRF44 | Brilliant Violet 650 |
| Live/Dead |  | Aqua Viability Dye |
| CD163 | GHI/61 | Brilliant Violet 605 |
| Ki-67 | B56 | PerCP-Cy5.5 |
| CD4 | L200 | Brilliant Violet 711 |
| CD20 | 2H7 | Brilliant Violet 421* |
| CD3 | SP34-2 | Brilliant Violet 421* |

*The gating strategy allowed use of this fluorophore for two different markers.
